# Winning! Election returns and engagement in social media

**DOI:** 10.1371/journal.pone.0281475

**Published:** 2023-03-01

**Authors:** Ernesto Calvo, Tiago Ventura, Natalia Aruguete, Silvio Waisbord

**Affiliations:** 1 Government and Politics, University of Maryland, College Park, Maryland, United States of America; 2 The Interdisciplinary Lab for Computational Social Sciences, University of Maryland, College Park, Maryland, United States of America; 3 Center for Social Media and Politics, New York University, New York, NY, United States of America; 4 UNQ—Universidad Nacional de Quilmes, Bernal, Argentina; 5 School of Media and Public Affairs at the George Washington University, District of Columbia, Washington, D.C., United States of America; University of Exeter, UNITED KINGDOM

## Abstract

This article analyzes social media engagement when elections are adjudicated to one of the contending parties. We extend existing models of *political dialogue* to explain differences in social media engagement (i.e. *time-to-retweet*) when users support the winner or losers of an election. We show that users who support the winning candidate are more engaged and have a lower time-to-retweet. We also show heterogeneity in Twitter engagement conditional on the number of followers, with accounts with more followers being less sensitive to the election result. We measure the effect of electoral adjudication using a regression discontinuity design, with estimates by winning or losing status, and for accounts with many followers (high authority) or with few followers (low authority). Analyses use Twitter data collected in Argentina (2019), Brazil (2018), the United Kingdom (2019), and the United States (2016).

## 1 Introduction: A tale of two elections

“It was the best of times, it was the worst of times, it was the age of wisdom, it was the age of foolishness, it was the epoch of belief, it was the epoch of incredulity” [1]. Election night, when one candidate is declared the winner of the electoral contest while other candidates recognize defeat, is a momentous occasion in democratic representation [2]. As the election is called to one of the parties or candidates, supporters rejoice or commiserate together. Winners celebrate and share social media content while losers quietly empty the scene [3].

How voters react when their preferred candidates win or lose an election has been extensively studied by scholars in political communication and comparative politics. Losing elections is associated with lower perceived regime legitimacy [4], lower satisfaction with democracy [5, 6], and lower political trust [7]. However, less is known about the effect of winning and losing on social media engagement [8].

In this paper, we show changes in Twitter engagement during election nights. We measure changes in the *time-to-retweet* before and after the election winner is called, testing for differences between users that support the winner and users that support the losers. We define the moment in which elections are called in favor of one of the candidates as electoral adjudication. The *adjudicator* may be an election authority, the media, or the candidates themselves. Once the election is adjudicated, voters accept an election outcome as definitive.

Twitter has become a most important platform for disseminating political content, with a large number of voters and politicians using it as a prominent source of information [9–11]. While findings on Twitter engagement will likely differ from other major platforms such as Facebook, WhatsApp, TikTok, or Youtube, our analyses provide persuasive within-platform variation in engagement when elections are adjudicated.

Our results show critical differences in the *time-to-retweet* when users support the winners or losers of an election. To understand these different levels of engagement, we provide further analyses comparing adjudication dynamics across two distinct dimensions: first, information drift, which describes the process by which information about winners and losers leaks before the adjudication. In a more formal definition, we use the term ‘information drift’ to describe the statistical effect of non-stationarity in a time series of Twitter engagement, where the moving average of our target variable is changing for reasons other than the sharp discontinuity of reporting an election winner. *Information drift* represents a challenge to our regression discontinuity design because the magnitude of the discontinuity depends not only on the intervention (e.g., the adjudication) but also on how much information has already leaked about the likely outcome. We use the term “information drift” to highlight that, as information about the likely winner ‘leaks,’ the magnitude of the discontinuity will change, a function of the moving average describing the users updating of new electoral data., second, network effects. Information drift describes the process by which information about winners and losers leaks before the adjudication and allows us to measure to which degree social media users adjust their behavior before elections are called. Network effects measures to which degree high-authority users with many followers differ in engagement from those users with fewer followers. Similar to recent studies on critical events and social media engagement [12], we describe a process in which high salience events increase engagement by infrequent users, generating a less hierarchical social media dialogue.

We conclude the article by exploring the role of emotions, in particular the use of uncivil speech, as a driver for this winner-lose gap in social media engagement. In a recent article, Liliana Mason argued that “Partisan emotions tend to arise in response to political actors or messages that have the power to affect the ultimate status of a person’s party—whether the party wins or loses [13]. Threats to a party’s status increase anger, while validation of the party’s status increases enthusiasm” [14]. Liliana Mason provides experimental evidence of an increase in anger when respondents perceive a threat to the status of their in-group (Page 5). Groenendyk and Banks [15] also show that partisans engage in political activity when strong emotions such as *anger* and *enthusiasm* are triggered. This line of research is increasingly being expanded to social media studies, and recent research has provided evidence that negativity and anger are key drivers for social media engagement, particularly in the context of politics and out-group polarization [8, 16, 17]. Therefore, our final analyses focus on the role of emotions on Twitter engagement at the time of adjudication, with particular attention to the patterns of toxic speech dissociating engagement among winners and losers during election night.

We test the proposed argument using a regression discontinuity design with *time-to-retweet* as our dependent variable. The analyses are conducted on Twitter data from four very polarized executive elections in the United States (2016), Brazil (2018), Argentina (2019), and the United Kingdom (2019). The case selection of these four elections considers critical traits that make them readily comparable. First, these are four countries with free and competitive democratic elections dominated by two leading candidates that concentrate most of the vote.

These four cases also display important structured variation in electoral rules, which affect how information about the likely winner and loser is disclosed to voters. In particular, we consider how “surprising” is the disclosure of information, which depends of the extent to which information about the likely outcome may be anticipated by the voters prior to the adjudication of the election to one of the parties or candidates. The variation in institutional design considers two extreme cases: a “sharp” adjudication in the United Kingdom, a non-federal country that sets a pre-specified time to call the election; and a “fuzzy” adjudication in the United States, a federal presidential regime whose Electoral College rules prevent voters from easily anticipating the likely winner for long periods of time.

We consider two intermediate cases, Argentina and Brazil, which are federal countries that publish election results over a relatively short (but not sharp) period of time, allowing voters to slowly update their expectations and alter their social media behavior. Results show politically meaningful differences across these institutional environments, moderating the engagement of those users that support the winners and losers of the election.

This article is organized as follows: we first describe a model of electoral adjudication that connects Twitter engagement with electoral adjudication. We then present the hypotheses of our study, which describe the reaction of winners and losers when the election is called as well as the different expectations for authority users with a large number of followers (top 10% quantile of followers). The third section describes the four case studies we analyze in this article: the United States (2016), Brazil (2018), the United Kingdom (2019), and Argentina (2019). In the fourth section, we describe extensions of our analyses to understand toxic speech among the losers of the election. Finally, we discuss the contribution of our findings to the extant literature and the limitations of our study.

## 2 Political dialogue and electoral adjudication: Hypotheses

In the last twenty years, political science scholars have established a robust literature showing that voters who support the winner(s) of an election report higher levels of trustworthiness, satisfaction with democracy, and perceived legitimacy [5, 7]. Micro-level studies of voters’ perception show changes in attitude before and after elections, with losers consistently reporting more negative views of democratic governance than those who supported the winners [6]. More recently, [3, 18] showed larger negative assessments among individuals who mistakenly expected their preferred candidate to win the contest (“surprised voters”). It is expected that this well-documented winner-loser gap will also affect Twitter engagement at the time the election is called for one of the contenders rathje2021out,justwan2018social. Therefore, following from this literature, our main hypotheses is that winners will be more engaged than losers in Twitter as they acquire information regarding the outcome of the election.

*H*_1_: Winners become more engaged than losers and, consequently, reduce *time-to-retweet* as credible information about the outcome of the election is reported.

Given that engagement is a function of how credible information about the outcome of the election is reported to the public, we consider two moderators of engagement: 1) information drift and 2) network effects. The former, information drift, is the result of voters being able to better anticipate the outcome of the election based on partial evidence. The latter, network effect, is the result of differences in information by network authorities (higher in-degree) compared to less connected nodes (lower in-degree). We describe these two moderators of user engagement next.

### 2.1 Information drift as a moderator of winner-loser engagement

Information drift is a function of the availability of credible and abundant data to anticipate the winner of the election before adjudication takes place. Such information drift can be estimated from observational data, providing researchers with evidence of changes in the odds of winning that result from differences in electoral institutions and rules. We expect the time-to-retweet to decline among winners at a faster rate than among losers prior to adjudication when electoral authorities report the election results over longer periods of time:

*H*_2_: Information drift, will be more pronounced in high information environments, under staggered elections rules, and less restrictive reporting laws, reducing *time-to-retweet* among winners prior to adjudication.

### 2.2 Network degree as a moderator of engagement

Small world social networks such as Twitter [19] are characterized by the average shortest distance between two vertices that “increase logarithmically with the number of vertices”[…]“The latter property gives the name small-world to these networks because it is possible to connect any two vertices in the network through just a few links” [20, pp. 11149]. As the election is adjudicated, the number of users (vertices) will increase more rapidly among the winners of the election and more slowly among the losers. While network authorities (users of high in-degree with many followers) will remain constant, the periphery of the winning community will grow at a faster rate. A winner’s “rising tide” will amplify the preferred content of the winners to a larger extent than for losers. Therefore, as the number of followers and the in-degree of a Twitter user declines, the adjudication effect will increase:

*H*_3_*a*: Electoral adjudication will result in lower time-to-retweet among the winners as the in-degree of the users declines (e.g., more active periphery).
*H*_3_*b*: Electoral adjudication will result in higher time-to-retweet among the losers as the in-degree of the users declines (less active periphery).

To simplify estimation, rather than estimating a regression discontinuity using a continuous interaction, we distinguish high authority users as those that are in the top 10% (top .10 quantile) in the total number of followers. Alternative specifications using the top .10 quantile in in-degree yield similar results.

## 3 The dynamics of adjudication

To test for the winner-loser gap in attention, we distinguish three different social media periods that structure Twitter engagement on Election Day: In the first period, there is a (i) steady level of Twitter engagement (*state of dialogue*) that is explained by the attention given to the election event by all users. In a second period, as the moment of adjudication approaches, there may be a pre-adjudication increase in information about the election result, where voters anticipate the likely outcome as a function of the disclosure rules of the electoral system. Finally, a third period begins with a (iii) discontinuity shock, where a credible announcement of the election result is followed by changes in Twitter engagement by the winners and losers (*post-adjudication*).

### 3.1 State of dialogue

Consider an initial state where an event is recognized as salient by all participants (*shared attention*) and there is no information about the likely tally of the votes. We define the moment prior to electoral adjudication as a *state of dialogue*, implying that all interested parties have the incentive to talk about the standing event whose outcome remains unresolved. As in [21], dialogue is defined narrowly as *individuals engaging on the same topic*, which does not require that they answer to each other’s Tweets. That is, we are not restricting “dialogue” to “conversation” as defined by Twitter, which links together replies and quotes to a primary tweet.

When a winner is called, (e.g., adjudication) supporters of the winner are granted *issue ownership* of the election [22]. Because *talking* about an issue or event raises its salience among voters [23], candidates are expected to talk about issues on which they are perceived to have an advantage [21, 24]. Extrapolating over from issues, when elections are called, *winning* decides which side owns the electoral race. Meanwhile, failure to adjudicate a winner, such as an outcome that is not recognized by one of the groups, will revert back to the *state of dialogue*, where all interested parties continue to talk about the election event at similar rates.

Before electoral adjudication, we expect dialogue to be solely explained by the overall salience of the election event [12]. More salient elections yield higher Twitter engagement (e.g., executive election) than less salient elections (e.g., midterm election), as it was described by [25]. Prior to adjudication, we expect differences in attention between supporters of all candidates will result from different priors for the likely winners and losers. As information of the election result increases, winners will increase Twitter engagement compared to the losers, *information drift*.

### 3.2 Pre- and post-adjudication

Adjudication is the moment when a candidate, party, or group is recognized as the winner of an *election* by an *adjudicator* that is universally recognized by all participants. An election may be called by any number of individuals and institutions, such as the winner, the loser, the media, an election authority, etc.

While failures in adjudication will result in higher Twitter engagement, with users reverting to the state of dialogue, the opposite is true about information drift. That is, information leakage will increase Twitter engagement among likely winners at a faster rate than for likely losers. Staggering election results, which allow voters to update expectations over time, provide an example of rules that facilitate information drift, energizing likely winners and silencing likely losers before adjudication is realized. Therefore, prior to adjudication, social media engagement is the result of shared attention [12], with supporters of the different candidates maintaining high levels of engagement.

**Fig 1:**
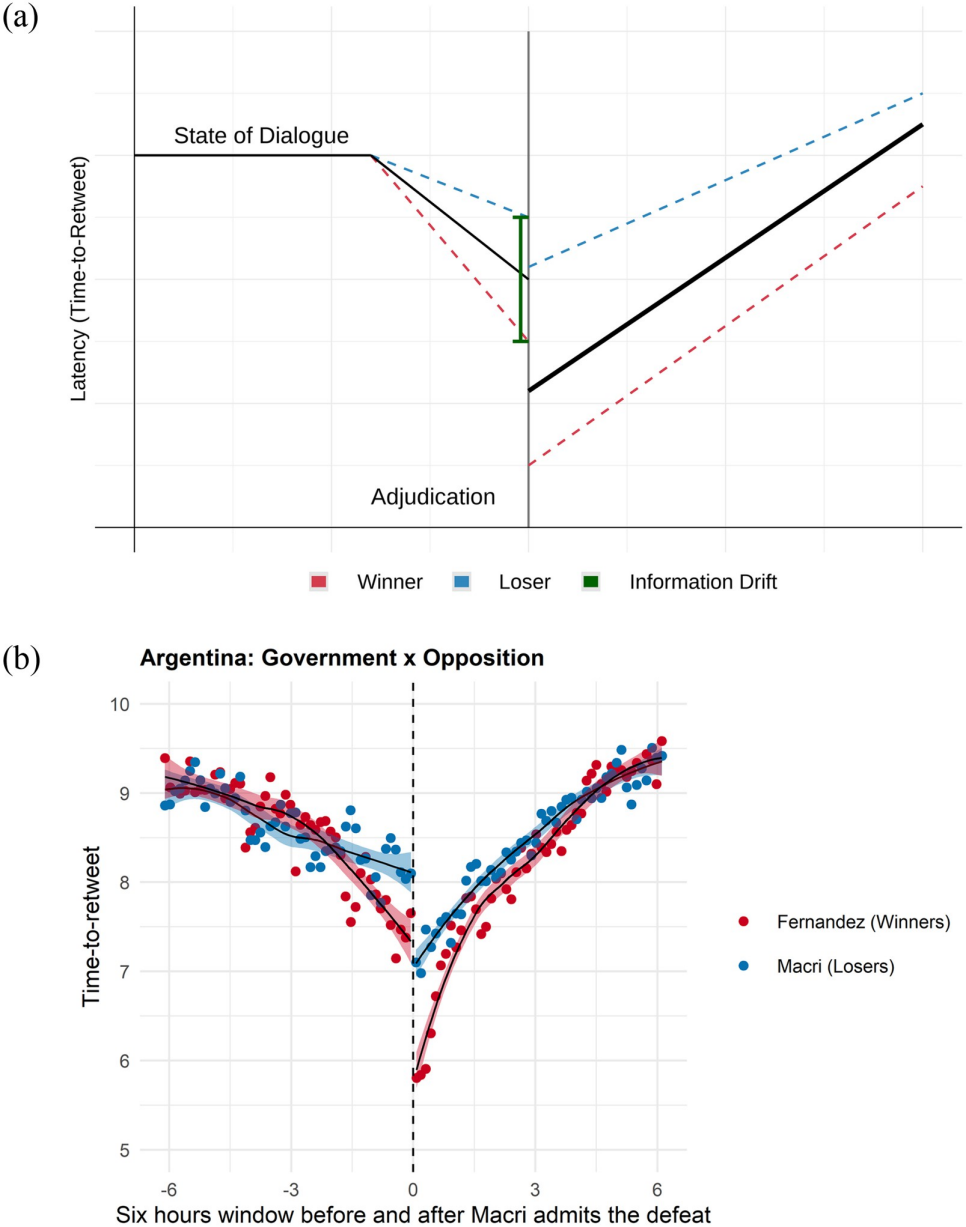
Adjudication and social media engagement. Fig 1(a) describes the expected increase in social media engagement, measured by faster Time-to-Retweet when electoral victory is adjudicated. Fig 1(b) describes the observed evolution of Time-to-Retweet in the observational data, Mauricio Macri’s defeat on October 11, 2019. (a) Theory, (b) Macri PASO Election.

Fig 1 summarizes the previous discussion. Prior to adjudication, users are in a *state of dialogue*, (a). As the election tally begins, users update their beliefs about the election outcome and increase engagement depending on their winner-loser status. *Time-to-retweet* declines at a faster rate for the winner, describing higher levels of Twitter engagement. In Fig 1(b), more engagement is described by a declining *time-to-retweet* in the y-axis.

Fig 1(a) also describes the effect of information drift on social media sharing, with likely winners increasing engagement at a faster rate than likely losers.

Upon adjudication, Fig 1(a) describes an expected discontinuity, with both winners and losers increasing their intent to share the results of the election (lower time-to-retweet). We expect a larger discontinuity among winners, controlling for the information drift that may decrease the value of adjudication. Finally, users will revert back to the initial state of dialogue as the salience of the event declines.

In Fig 1(a), the green vertical line before adjudication describes the difference between the likely winners and losers, the *total information drift*, just before adjudication. The vertical yellow solid line immediately after adjudication, on the other hand, describes the differences between the winners and losers when election results are made public. We label the discontinuity after adjudication as the *total adjudication effect*.

Each of these different parameters can be empirically estimated and compared across election events, allowing us to understand how accepted the adjudicator is (divergence in dialogue), how sharp is the disclosure of the election results (low information drift), as well as the magnitude of disaffection on among losers (total adjudication effect). Each of those parameters of interest, therefore, allows researchers to better understand social media behavior on Election Day.

Fig 1(b) provides a vivid example of our model of adjudication, with Twitter data collected during the electoral loss of President Mauricio Macri in Argentina, on October 11 of 2019. Fig 1(b) evaluates adjudication, with a window of 6 hours before and six hours after President Macri admits electoral defeat.

We may use Fig 1(a) to understand the behavior of the data in Fig 1(b). On the left side of Fig 1(b), we see a slow decline in time-to-retweet that is the sole result of increased salience. Users that are aligned with the future winner (Fernandez) or loser (Macri), increase dialogue and engagement as we approach adjudication. One hour prior to adjudication, however, we see evidence of information drift, where the soon-to-be winners and losers update their beliefs, and their time-to-retweet diverges. The PASO election of 2019 provides a narrower drift than other election nights, as President Macri recognized defeat at 9:20 PM before any electoral results were disclosed by the Electoral Authority (DINE).

As President Macri recognized defeat, we see a sharp discontinuity among winners and losers, with a larger drop in time-to-retweet among those that celebrate (*enthusiasm*) and a lower discontinuity among the losers (*disaffection*). Of course, this is a relatively trivial result, as we always expect enthusiasm among winners and disaffection among losers. However, we call the attention of readers to the value of understanding the magnitude of the information drift and the importance of the adjudication effect, which are of extraordinary comparative value to understanding information propagation and dialogue in social media.

Finally, over time, salience declines as well as the *enthusiasm* or *disaffection* by users, which prompts us back to a state of dialogue, subject to the overall salience of the event after adjudication and to the circadian rhythm of social media usage.

## 4 Four elections: Johnson, Macri, Bolsonaro, and Trump

We compare electoral adjudication in four different elections in the United Kingdom, Argentina, Brazil, and the United States, with attention to two different groups of users (high-level authorities and low-level authorities) that speak to the relationship between network structure and dialogue. In the first two cases, we will show there is little in the way of information drift. In the last two cases, Brazil and the United States, staggered disclosure of electoral results provide for more significant information drift. In all four cases, we compare (and explain) differences in adjudication, the total information drift, and the total adjudication effect.

### 4.1 Four election nights

We ordered the four election nights to reflect the insights of our theory, ordered from the one with the lowest information drift–the United Kingdom–to the one with the longest information drift–the United States.

The United Kingdom, Argentina, Brazil, and the United States, held elections for the Head of Government on December 12 of 2019, October 11 of 2019, October 7 of 2018, and November 8 of 2016, respectively. Arguably, the UK election of 2019 was among the most meaningful elections in a generation, as it was expected to ratify or dispute the Brexit referendum and grant or deny Brexit negotiating authority to Boris Johnson. As important for this article, as a result of the *Representation of the People Act 1983*, enforced by the *Office of Communications (Ofcom)*, all media outlets are prevented from publishing news that forecast the result of the election and an excellent official exit poll provides rapid adjudication of victory to candidates on election night.

In the cases of Argentina and Brazil, we selected the first *round* of the Presidential Election, when voters have limited information on the likely outcome of the race. The first round of the Argentine presidential election of 2019 was the *Open and Simultaneous Presidential Primary Election*, known by its acronym *PASO*. This is a compulsory national election where all adult citizens are required to cast a vote. Different from the second round of October 27, the *PASO* provides a mechanism to select presidential nominees. However, all important Argentine candidates ran unopposed in 2019, in what was *de facto* the first of a three-round presidential race.

The timeline of the Argentine election was short and relatively simple, with voting ending at 6PM and results expected to be reported starting at 9PM by the National Direction of Elections (DINE). On election night, however, a slower than usual tally of the votes meant that by 9:20 PM the dashboard of the election authorities was still showing no data. At 9:32 PM, President Mauricio Macri recognized defeat still with no electoral results being reported to the public. Within the hour, the official numbers began to be reported to the public.

The first round of the Brazilian presidential election on October 7 of 2018 is also a compulsory election where all adults are required to vote. As in the case of Argentina, failure to vote is met with a legal fine or the requirement to justify a no-vote, something that will often consume a fair amount of time. Results of the Brazilian election are known within three hours of closing of the ballot boxes, as a single e-vote device is used in all 32 states. The timeline of the Brazilian is even shorter than in Argentina, with voting ending at 6 PM and partial results expected within the hour. On election night, a notice of a convincing victory by Jair Bolsonaro was reported immediately after the closing of the ballots. Just two hours later, at 8:02 PM, with 96% of the votes tallied, Bolsonaro was leading the second most voted candidate, Haddad, by almost twenty points. As in Argentina, the race was defined by a significant larger margin than anticipated by most pollsters. Finally, at 22:04 PM, Bolsonaro gave a victory speech to his supporters.

The fourth and final election, the United States Presidential Election, is a single-round contest where all registered voters have the option to cast a vote. The winner is decided by a majority of electoral college votes, with reporting taking place over many hours, as each State reports its own results. A long tally with staggering results allows more significant information drift, compared to the cases of Argentina or Brazil. On November 8 of 2016, critical battleground states were reported over the course of several hours, beginning with the critical victory of Trump in Ohio at 10:39 PM Eastern Time, followed by reported victories in Florida (10:53 PM), North Carolina (11:14 PM), and Pennsylvania (1:35 AM). Finally, at 2:35 AM, Hillary Clinton called Donald Trump to congratulate him on his victory, which was given ample space in the media. Different from the cases of Argentina and Brazil, the US reporting of election results is considerably longer, allowing voters to update their expectations on the likely winner. As we will show, this is clearly visible in the increasing difference in the time-to-retweet of Democrats and Republicans on Election night.

### 4.2 Data collection

All data for this project was collected using a single Twitter Academic account. The current license was extended on January 29, 2021, with access to the historical repository under the registered project and Twitter App “Winning! Electoral Adjudication and Dialogue in Social Media”, approved by the *Twitter Dev Team*. Human Subjects and Ethics approval was requested and the determination of EXEMPT STATUS was granted to the iLCSS repository by the University of Maryland Institutional Review Board (IRB) on November 18, 2021. The project approval is registered under the identification code 1836757–1, certifying the ethical use of all data collected using Twitter’s V1 and V2 API.

To analyze adjudication and dialogue, we followed the same procedure in all four countries. First, we collected a large sample of tweets from the beginning of Election Day until one day after the election (6,7 million tweets from the UK, 6,7 million tweets from Argentina, 4.9 million tweets from Brazil, and 5,2 million tweets from the US). We collected data accessing both Twitter Streaming and Restful APIs. The latter allows the public to access a temporary repository of tweets that includes a large sample of all tweets published during the week prior to the query, while the streaming API lets users capture tweets in real-time. We use keywords that include the main candidates’ and main parties’ names as well as keywords related to the election. We use the Python base program *Twarc* to access the APIs. For our data collection, we use the queries with the names of the main national candidates in each of the four countries.

We filtered singletons (one-time users), selected only those tweets posted in the country’s primary language, and retained the first connected cluster of each country. In all four cases, these primary connected clusters contained the main political networks that were politically engaged during the election. For community detection, we implement a two-step approach: first, we implement a computational community detection of the tweet networks via *random.walk*, assigning all accounts to unique communities [26]. Second, we sort the accounts in each community by their in-degree and the top tweets by the number of retweets and proceed to manually inspect each community to label the main political groups. In all four cases, the largest communities correspond to the top two candidates in the respective election. Other relevant communities are shown in section 1 of the S1 File. The S1 File also provides two more pieces of information to validate our community detection choices: a) the list of the top 30 users in each of the communities and b) the maximally discriminant hashtags that circulated in each community to show readers the unique content that circulates across each group. Both pieces of information were qualitatively validated by the authors.

While we use the full primary connected network to estimate the communities of the politically engaged users on Twitter, the analyses of engagement use a 12-hour window, six hours before and six hours after adjudication. Therefore, we use all the network data to identify the community of the users, benefiting from a larger sample to derive the properties of the network, but study political dialogue at the time of adjudication (See the S1 File for further details on the countries’ networks).

### 4.3 The statistical model

To determine the effects of event adjudication, we use an interrupted time series analysis, a variety of regression discontinuity designs (RDD) in which the running variable is time [27]. Twitter data is ideal for our approach because of the granularity and high frequency of tweets. Our primary parameter of interest is the change in social media users’ behavior upon the adjudication.

The RDD models use *time-to-retweet* as dependent variable. This variable captures changes in social media engagement on users’ behavior before and after adjudication and uses the number of seconds elapsed from the time a tweet is posted by a user to the time it is retweeted by a second user. To normalize the variable, we use the log of time-to-retweet in the statistical models. Because users can retweet messages created at any point in time, the decision to log the time-to-retweet adjusts the fact that the dependent variable is right-skewed with some extreme outliers on the right tail. For example, if a user *i* retweets a tweet sent by user *j* x minutes after the message was sent, our dependent variable will be 60*x seconds for the observation *tweet*_*ij*_. Our unit of analysis is, therefore, any retweet collected using the methods described above, from which we retrieve information about the time, the author of the tweet, and the user who retweeted the original message. Previous research has extensively used *time-to-retweet* to understand heterogeneity in content propagation, news sharing, and activation on Twitter [28–30]. The time of the event adjudication is the cut-off of the discontinuity regression model. Our parameter of interest approximates the changes at the time of the adjudication, when the cut-off is equal to zero, on *time-to-retweet*. We used a set of news reports and qualitative analysis by the authors to estimate the precise moment of adjudication for each case.

To estimate the models, we follow the recommended setting of using non-parametric local linear regression (LLR) to approximate the treatment effect at the cutoff point [31, 32]. We employ a local polynomial with one degree to fit two separate regression functions above and below the cutoff Adjudication, with the treatment effect set as the difference in the limits of the cutoff. We employ triangular kernel weights and employ a data-driven search to select an optimal bandwidth for the estimation. To address potential bias on the treatment effects due to approximation errors, we report the robust treatment effects and confidence intervals developed by [32]. We estimate all models using the R package *rdrobust* developed specifically to implement recent developments in the regression discontinuity designs literature [33].

Regression discontinuity models assume that effects are continuous at the cutoff. When dealing with time as a running variable, the continuity assumption requires that no omitted variable that systematically affects the outcome—*time-to-retweet*—also changes upon the adjudication. Given that we have the precise minute when Adjudication was granted and consider data only six hours around the cutoff, it is reasonable to assume that this assumption holds. Although winners and losers in each country have important socio-demographic differences, for example age, income, or education, all these between-group differences do not change upon the adjudication. Therefore, our point estimates are causally identified since our target parameters are all estimated within groups. In addition, network dependency across the observations is also sorted out due to our within-community strategy (See S1 File for a set of tests to verify the continuity assumption). The granularity of the data, together with the precise measurement of the event, makes the identification strategy highly plausible. The S1 File provides a set of tests to verify the continuity assumption, including placebo checks with the running variable and methods to estimate inconsistent patterns of anticipatory behavior among the users before the adjudication. Overall, the results ensure the internal validity of the RD design.

### 4.4 Results

Using the data and the specification described above, we estimate twelve regression discontinuity models. The discontinuity parameters of interest are measured at the time that the early count of the UK is reported by the media (*December 12, 2019, at 5:00 PM, local time*); at the time that Mauricio Macri acknowledges defeat on national television in Argentina (October 12, 2019, at 11:21 PM, local time); at the time the media communicates that exit-polls show a conclusive victory of Jair Bolsonaro in Brazil (October 7, 2018, at 7:04 PM, local time); and when the media reports that Hillary Clinton called Donald Trump to congratulate him on his victory in the United States (November 8, 2016, at 2:35 AM, local time). In all four cases, those are the defining moments of adjudication as they make clear to voters who is the election winner. These four times also coincide with the highest level of Twitter activity on Election Day.

**Fig 2:**
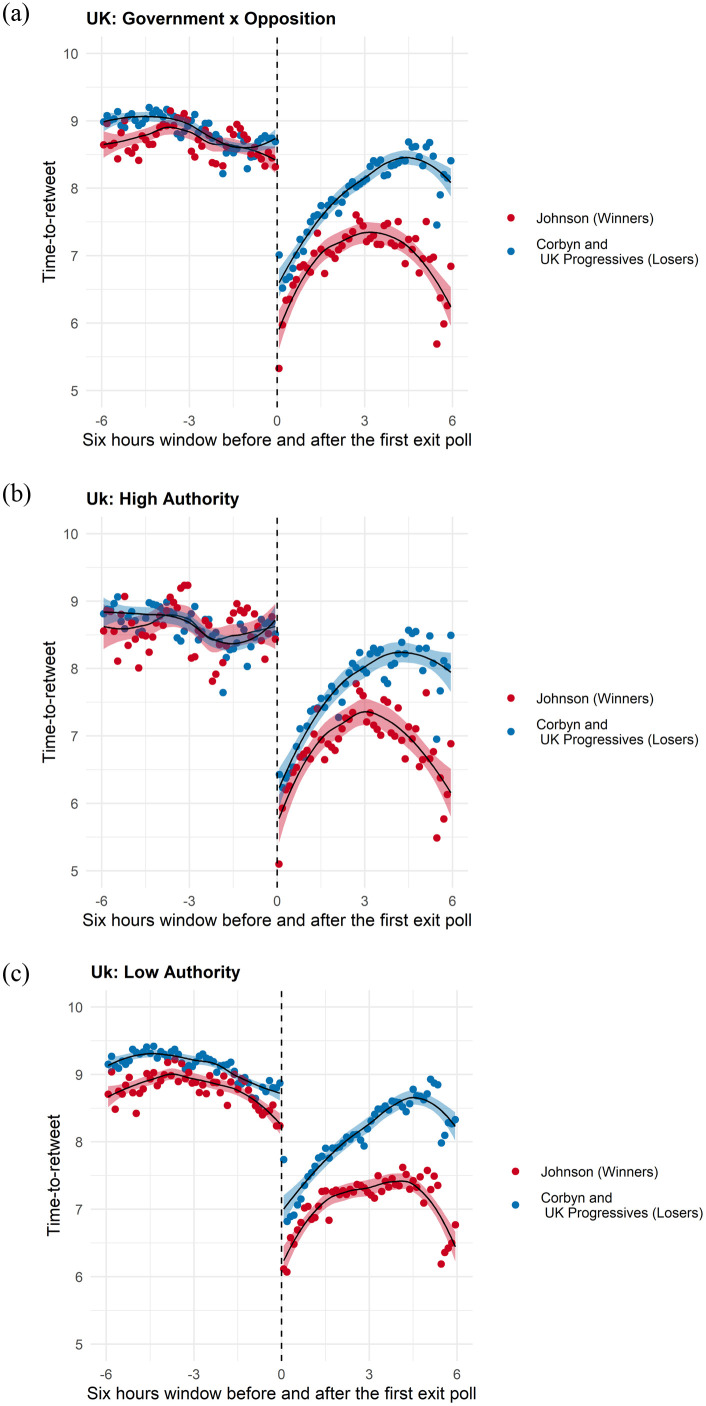
Time-to-retweet in the UK election. (a) Winners vs Losers, (b) High Authority Users, (c) Low Authority Users.

**Fig 3:**
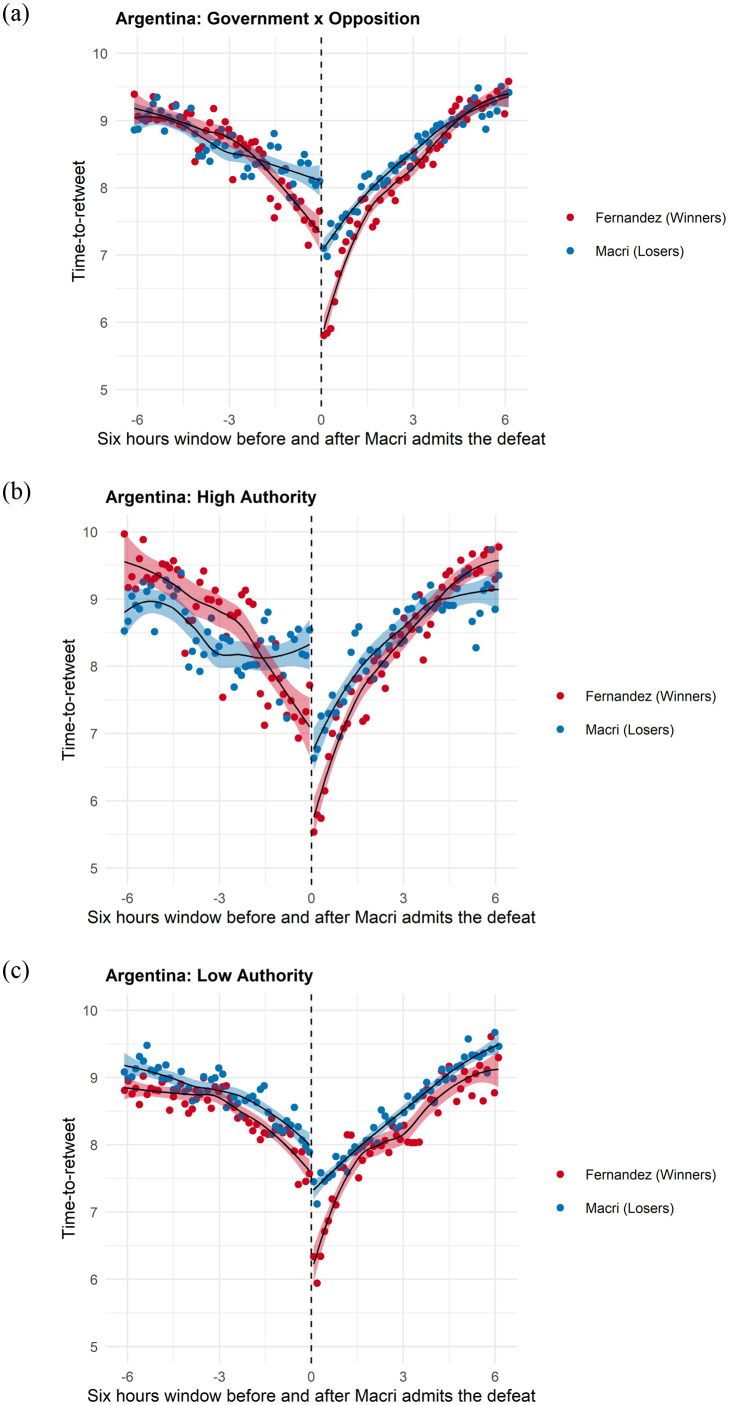
Time-to-retweet in the argentine election. (a) Winners vs Losers, (b) High Authority Users, (c) Low Authority Users.

**Fig 4:**
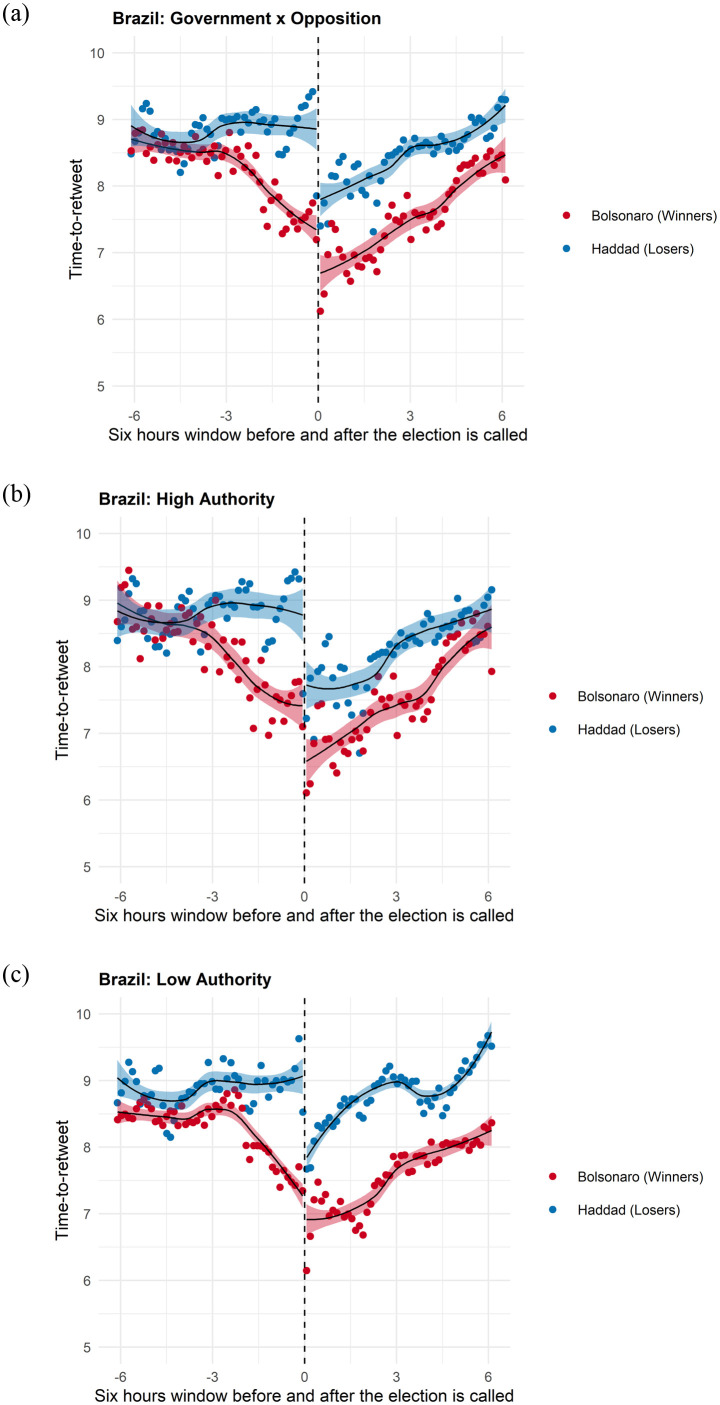
Time-to-Retweet in the Brazil Election. (a) Winners vs Losers, (b) High Authority Users, (c) Low Authority Users.

**Fig 5:**
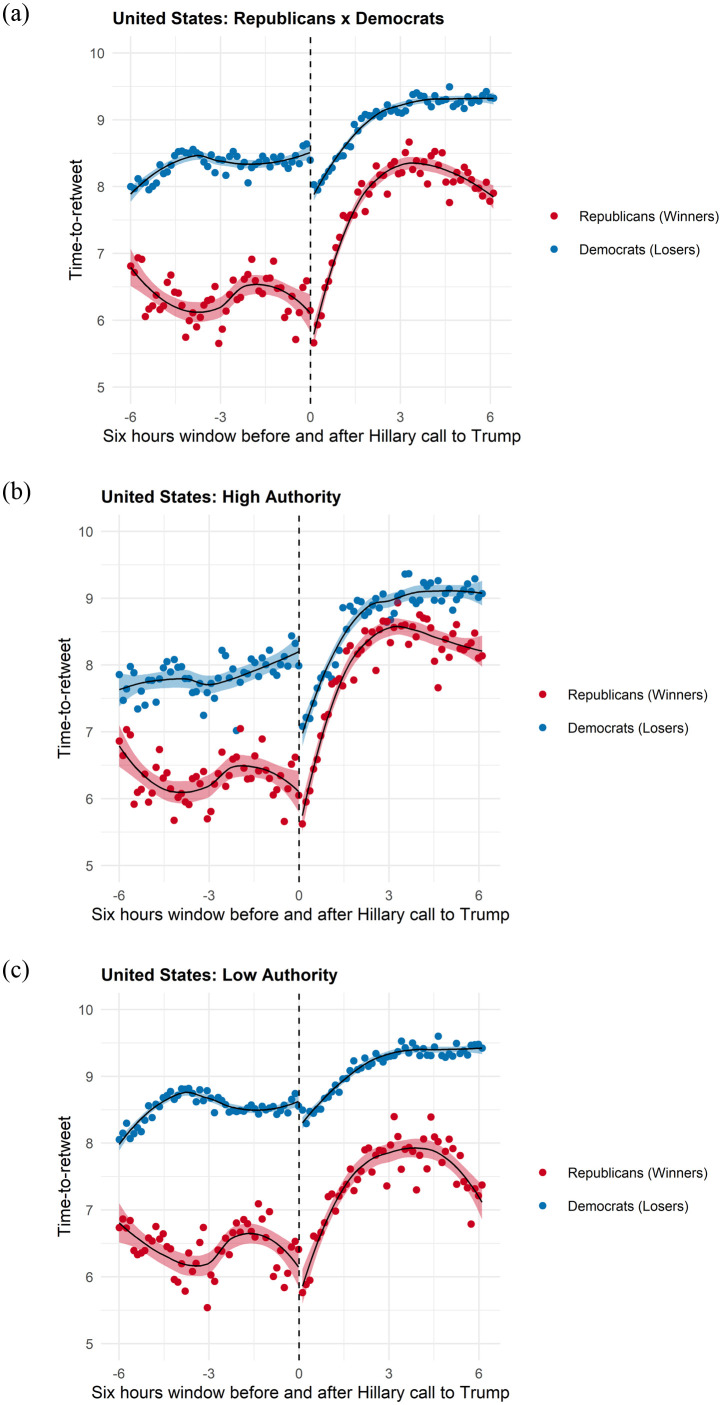
Time-to-Retweet in the US Election. (a) Winners vs Losers, (b) High Authority Users, (c) Low Authority Users.

Figs 2–5 provide vivid images of electoral adjudication in all four countries. The vertical axes report the log of the time-to-retweet, with lower values indicating that users are more engaged (lower time-to-retweet). The horizontal axes have a range of twelve hours, six hours before and six hours after adjudication. We use a LOESS smoother with separate lines before and after adjudication. To make visualization easier, we binned the data over time. Readers can readily observe how the behavior of users emulates (and how it differs) from the theoretical model in Fig 1(a).

Let us first consider Figs 2 and 3. They present the overall adjudication effects on the left plot, Figs 2(a) and 3(a); estimates for tweets published by high in-degree users(the top 10% quantile log-median number of followers), Figs 2(b) and 3(b); and by low in-degree users (below the 10% quantile in the log-median number of followers), Figs 2(c) and 3(c).

We ordered our cases from those with the lowest information drift (the UK and Argentina) to those with the highest information drift (Brazil and the US). Fig 2(a) shows a pattern where shared attention is increasing before adjudication (declining slope), and winners and losers are equally engaged (state-of-dialogue) *H*_1_. We also document a large discontinuity for the UK case, with Johnson supporters more active than Corbyn supporters upon adjudication *H*_3_*a*. An interesting feature of the data is that attention is larger among low-authority supporters of Boris Johnson even before adjudication, as described by the “rising tides” logic [12].

Fig 3(a) is identical to Fig 1(b) in the theory section, with a very small information drift and a sharp discontinuity at the time that Mauricio Macri acknowledges defeat in the PASO election. As noted in our prior discussion of this graph, electoral results had not been formally relayed by the National Electoral Directorate (DINE), which resulted in continued social media dialogue until half an hour before adjudication. As the campaign of the opposition candidate Alberto Fernandez begins to report that they have won convincingly, users that support him begin to more actively tweet messages and retweet each other more. The information drift of the last half hour is then followed by a large adjudication effect at the time of Macri’s news conference.

Fig 3(b) and 3(c) show similar behavior, with similar information drifts before and after adjudication. However, it is worth highlighting how high in-degree users of the losing community (blue line) have lower time-to-retweet than low in-degree users of the losing community, as expected in *H*_3_*a*. Readers can appreciate that in the initial *state of dialogue* shown in Fig 2(b), authorities supporting Macri garner more engaged responses than those of Fernandez, as expected in *H*_3_*b*. Meanwhile, the opposite is true among low authority users in Fig 2(c), as expected in *H*_3_*c*. In other words, low-degree users are more engaged with each other among the winners and less engaged among the losers. This feature of the graphs speaks directly to differences in social media networks that will be reported and discussed in Table 1 later in this section. In all, engagement is more dependent on high in-degree nodes (authorities) among losers and more reflective of low in-degree enthusiasm among the winners. The result is engagement that is more hierarchical among losers and more horizontal among winners.

**Table 1:** Adjudication in four cases: Information drift and adjudication effect.

	UK		Argentina		Brazil		United States	
Condition	Informational Drift	Adjudication	Information Drift	Adjudication	Information Drift	Adjudication	Information Drift	Adjudication
Winners x Losers	−0.46	−1.92	−0.46	−1.32	−0.76	−1.45	−1.99	−2.15
High Authority	−0.51	−0.12	−0.57	−1.01	−0.42	−1.40	−1.84	−1.34
Low Authority	−0.60	−2.29	−0.52	−1.36	−1.67	−1.84	−2.20	−2.69

The Bolsonaro election provides an example of an election that allows for more information drift before adjudication, as results of the election at the state level were reported to the public for over two hours. These results allowed voters to update their predictions about which candidates, aligned or not with Bolsonaro, were winning the sub-national elections. With a higher information drift, we also observe a smaller adjudication effect in each of the communities compared with the UK and Argentinian cases. It is interesting to know that the state-of-dialogue that precedes adjudication remains almost flat until the closing of the voting places, which happens almost three hours before the adjudication of the election. Fig 4(a) shows how, immediately after that, the pro-Bolsonaro users begin to engage while the losers disengage, therefore, providing us with a clear example of information drift. A small up-swing three hours prior to adjudication, when voting ends, shows the immediate effect of the “*boca da urna*” that is reported by the media, indicating a likely victory by Bolsonaro.

As in the case of Mauricio Macri, Fig 4(b) and 4(c) show higher sensitivity among low-authority users, who more readily disengage when losing and more actively retweet each other when wining. As in the case of Mauricio Macri, we can visually observe the network of the losing candidate as becoming more hierarchical, while the opposite is true among supporters of the winner.

Finally, results from the US election provide consistent evidence of a long adjudication cycle, with staggering results that allow voters to constantly update the winner of the race. The lengthy process of counting votes in the United States allows both communities to diverge slowly. Indeed, the state-of-dialogue is outside of the six-hour window, and the ebbs and flows of the State results that are reported to the public explain smaller shifts in engagement as we approach adjudication. Once adjudication takes place, however, we can see a rapid decline in engagement.

A result that is worth highlighting is that, different from the Argentine and Brazilian cases, the winner and loser communities never fully return to the state of dialogue in the US and in the UK. As we described earlier, this is likely due to the fact that both the UK and Trump elections were ones that provided a true final determination, as in both Argentina and Brazil, the winner of the election still had to win a second time. Both Alberto Fernandez and Jair Bolsonaro would win their next race comfortably, closing the election cycle in their respective countries.

**Fig 6:**
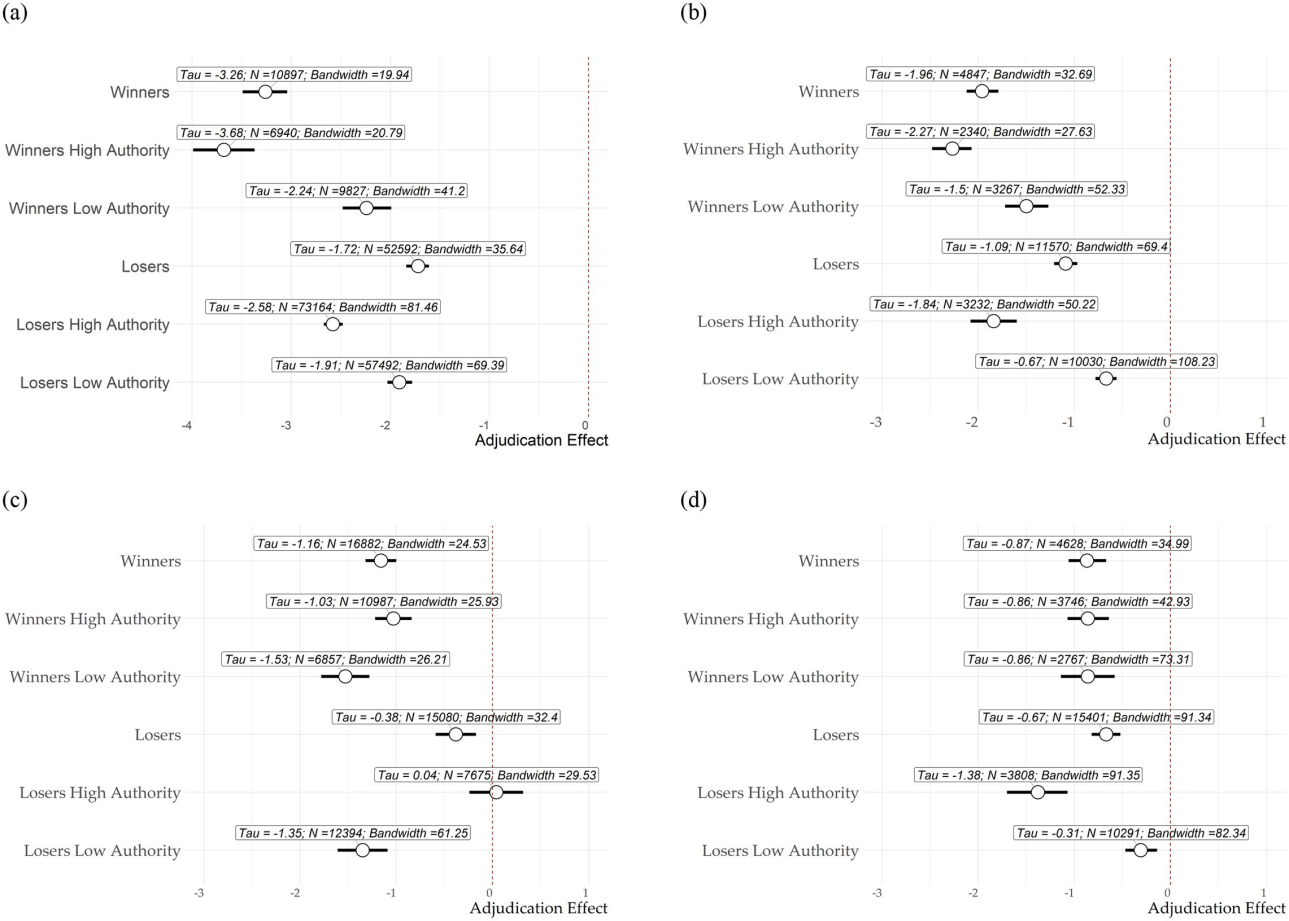
Adjudication effect. Results at cutoff estimated with local linear regression with triangular kernel and MSE-optimal bandwidth. The figure reports 95% robust confidence intervals for the point estimates [32]. (a) UK Election, (b) Argentina Election, (c) Brazil Election, (d) US Election.

Next, we present the point estimates for the regression discontinuity designs relying on [32]. The models present robust point estimates and confidence intervals and use the data-driven bandwidth selection method proposed in [32]. While Figs 2 to 5 introduces the reader visually to our main results, Fig 6 provides a precise interpretation of our findings. As in Figs 2 through 5, winners exhibit larger treatment effects on all four elections compared to losers. Electoral adjudication increases engagement among winners and reduces engagement on election-related topics. Results show, as expected that high in-degree authorities exhibit greater treatment effects for winners and losers as well in the majority of the four election cases, with the exception of the Brazilian case in which authorities among the losers show no effect. The effect is larger, in particular when information is scarce and drift is not observed before adjudication (UK and Argentina).

Table 1 presents the numerical results for the information drift and adjudication effects across all four cases. The quantities are estimated using the parameters retrieved from the twelve regression discontinuity models. As it was described in Fig 1(a), we measure information drift as the difference between winners and losers on the left side of the cutoff. By contrast, we measure adjudication as the difference between winners and losers on the right side of the discontinuity. A greater negative information drift indicates that losers are disengaging before adjudication. Meanwhile, a greater negative adjudication effect measures the difference between winners and losers upon adjudication.

Results show a much larger information drift in the elections won by Trump (-1.99) and Bolsonaro (-.76) and a much smaller information drift in the elections won by Johnson and Fernandez (-.46). As discussed earlier in this article, the difference in information drift in the US and Brazil is due to the staggered reporting of the results. Meanwhile, both in the UK and in Argentina, election results were reported within a very short period of time. Another interesting result reported in Table 1 is that the overall adjudication effect is larger in the United States and in the UK, which held conclusive elections. By contrast, effects were smaller in Argentina and Brazil, which still had a general election (Argentina) and a run-off (Brazil).

More important, in all four cases, we see that the total adjudication effect is significantly larger among low authority users and smaller among high authority users *H*_3_*b* and *H*_3_*c*. This is reflective of the more hierarchical nature of dialogue among losers and the more horizontal dialogue among winners. In other words, upon adjudication, disengagement is more prominent within low-authority losers and in the periphery of the networks, while the high-authority users overall keep similar levels of engagement. The largest network effect is in the UK, where the adjudication is orders of magnitude as large among low authority users. While the proportional network effect in Argentina and Brazil is similar, the absolute value is larger in Brazil.

## 5 Extensions: *Toxic* dialogue and adjudication

The analyses of the previous section introduced readers to four cases of electoral adjudication. We described the effect of adjudication on Twitter engagement, which increased among winners at a faster rate than among losers. We argue that this difference was driven by *enthusiasm* and *anger*, respectively. Overall, our results show that as elections are called, users who support the winning candidates display a lower time-to-retweet. The opposite is true among users who support the losing candidate, who were less engaged and displayed a higher time-to-retweet. In this section, we test for this mechanism via a study of toxicity in the corpus of Tweets at the time of adjudication expanding on previous literature on the role of emotions on political participation, including social media engagement mason2016cross, banks2014anger, suhay2018polarizing, rathje2021out.

We test for the role of emotion on Twitter engagement at the time of adjudication. To do so, we take advantage of recent developments in natural language processing (NLP) that measure toxicity in written speech. As in the previous section, we restrict our analyses to the six hours before and after adjudication in the four elections. We use Google’s API *Perspective*, a content moderating tool that is the industry’s standard for the automatic detection of toxic content in written comments. Perspective uses a convolutional neural net model to score the toxicity of input text in a corpus of documents. *Toxicity* is defined as “a rude, disrespectful, or unreasonable comment that is likely to make one leave a discussion.”. Google’s *Perspective* is widely used both in industry and by scholars in social media studies, including research that identifies toxic comments on streaming Facebook chats during political debates [34, 35], to classify YouTube comments made by politically engaged users [36], to classify Twitter’s uncivil political discussion [37], and to understand toxicity in Facebook and Reddit pages [38].

Recent analyses have shown comparable performance between the off-the-shelf API *Perspective* and the manually validated dictionary methods developed by experts with the specific purpose of identifying toxic and uncivil content [39, 40]. [38] find that *Perspective* generally outperforms single human labelers, providing an interesting combination of accuracy and efficiency. While it is true that automated classifiers will be less than perfect, *Perspective* high accuracy, low training costs, and comparability between different datasets as used in this project, make Perspective a cost-effective and highly replicable off-the-shelf computational tool to score toxicity levels across multiple datasets.

Fig 6 plots the *toxicity* scores retrieve from Google’s *Perspective* before and after the adjudication of the election in all four cases. The Perspective API provides each comment with a score from 0 to 1 corresponding to the probability the comment has the given attribute. We dichotomize this score and consider all comments with a score above .5 as having the attribute and below .5 as not having the attribute. The API Perspective is built as a classification algorithm, where the score is the likelihood of a comment being classified as “toxic”, and not a continuous measure. The decision to dichotomize the toxicity scores comes intuitively from the nature of the measure.

**Fig 7:**
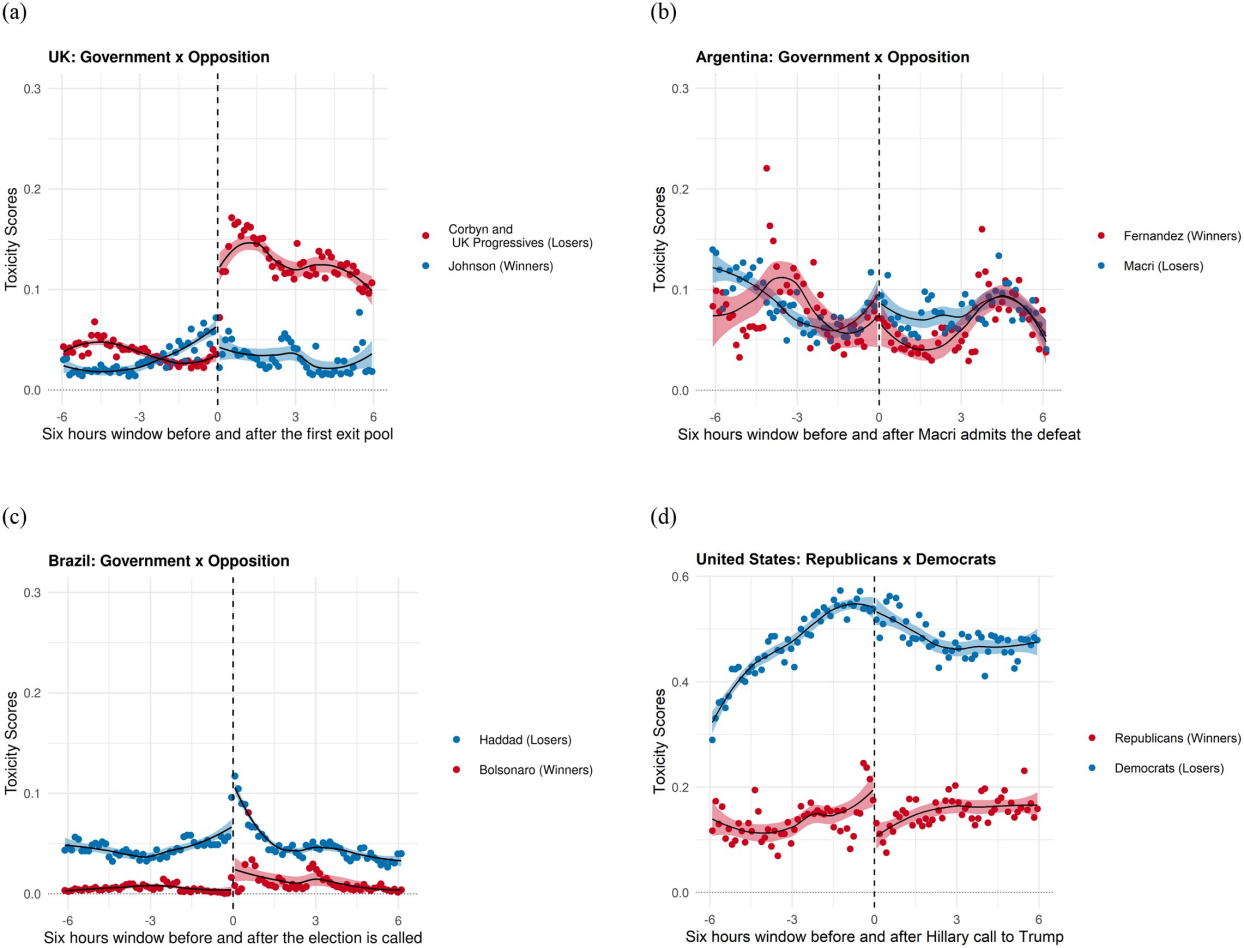
Toxicity scores of all the four election nights. (a) UK Election, (b) Argentina Election, (c) Brazil Election, (d) US Election.

Let us begin by discussing the results of the United Kingdom, which are textbook examples of partisan anger triggered by a negative election result. As it was described previously, the UK election has disclosure rule that produces little information drift prior to adjudication. Therefore, supporters of the losing party that remain engaged after the adjudication have little time to adjust their expectations. At 9PM, when Labor supporters are informed of the outcome of the election, the level of toxicity scored by the *Perspective* API increases fourfold, from less than .03 to over .13. By contrast, the Tweets of Conservative users who support Boris Johnson see almost no change in their average score. Previous research has shown that the 2016 Presidential election was at the time, the most negative election on the record [41], with a high prevalence of uncivil comments towards both candidates and, in particular, toward Hillary Clinton [42]. Toxicity scores in the United States align with prior research and complement differences in the engagement of winners and losers described in the previous section. Adjudication of the US Presidential winner was preceded by a lengthy tally over multiple States, allowing voters to slowly update their expectations about the likely winner. Fig 7 is revealing, showing a small decline in the toxicity scores of the winners that is met with increasing toxicity scores among supporters of Hillary Clinton. As shown in Fig 7, the difference in the probability of observing a toxic tweet grows monotonically as we approach adjudication. While the average probability of a toxic tweet among Republicans was close to .15, this value was close to three times higher among Democrats. The strong information drift makes adjudication effects for the winner negligible, yet it is also telling the sharp discontinuity in toxicity among Republicans at the time of adjudication.

Toxicity estimates for Brazil are supportive of an adjudication effect but less dramatic than in the United Kingdom and the United States. As the election tally is communicated to voters, the losers decrease their engagement (previous section) and share content that is less civil. The increase in toxicity among supporters of Haddad in Brazil is considerable larger than among supporters of Bolsonaro. However, results also show systematic differences in toxicity across the two groups both before and after adjudication.

Finally, findings in Argentina are inconclusive and toxicity scores are also considerable higher prior to adjudication. While there is a statistically significant difference in toxicity between the winners (Fernandez) and the losers (Macri) in the 2 hours post-adjudication, there is no statistically significant within-group discontinuity at the time that candidate Mauricio Macri recognizes defeat.

As described by [43], online toxicity is a multidimensional and context-sensitive behavior, which is moderated by information shocks but also by linguistic and cultural differences in social media uses and customs. While the results in the United Kingdom and in the United States align very well with the proposed theory, there is likely unmeasured heterogeneity in the data to be explained by factors other than the adjudication effect. Similarly, we suspect that the high heterogeneity observed in the toxicity scores of Brazil and Argentina reflects linguistic, cultural, and partisan differences that make statistical discrimination more difficult. While it would be tempting to consider the US, UK, and, to a lesser extent, Brazil as successful tests of our toxicity argument, it would be improper to explain away the Argentine null findings. Therefore, we consider that more research is needed to conclusively link toxicity and the winner-loser gap. Our analysis provides some new-sounding dynamics about changes in uncivil discourse in social media.

## 6 Discussion

In this article, we model electoral adjudication and dialogue in social media using data from Twitter. We focus on the moment in which one party is recognized as the winner by an adjudicator and test our expectations on four recent elections. After elections are called, losers reduce their Twitter engagement and use language with more prevalent toxic content. Winners, on the other hand, increase engagement and see a surge of dialogue in their periphery.

The model of electoral adjudication and dialogue proposed here has clear theoretical implications for scholars interested in social media engagement. Prior to adjudication, we describe similar engagement across communities. As users update their expectations, we describe an information drift where likely winners already increase their overall engagement.

We explain the observed engagement by winners and losers as the result of differences in enthusiasm and anger after they are exposed to information that confirms or rejects their expectations about the election. Differences in institutional rules moderate the behavior of users, as they allow information about the likely result to leak at different rates. More porous rules, we argue, explain more noticeable information drift between the winners and losers prior to adjudication.

### 6.1 Contributions to the existing literature

Our findings contribute to three well established theories on *political dialogue* [21, 24], *critical media events* [12, 44], and the *winner-loser gap* in election studies [2, 5, 45].

Existing theories of issue ownership and political dialogue purport that candidates should never “talk to each other” but rather that they should “talk past each other” raising the issues that these parties own [21, 24]. For example, republicans should talk about taxes and Democrats about entitlements. Labour candidates in the UK should talk about employment, while the Conservatives focus on crime. As salience increases, however, dialogue emerges. Candidates talk “past each other” on low-salience issues or events, but campaigns are forced to present competing narratives when salience increases because failing to address important issues is evidence of *tone deaf* or out-of-touch politics. After a major economic crisis, everyone talks about the economy. After 9/11, everyone talks about terrorism.

In political dialogue models, issue *advantage* and issue *salience* jointly determine the extent to which parties engage in political dialogue. However, the existing literature understands *issue ownership* or *issue advantage* as a performance trait that is acquired over time (For an excellent analysis of changes in party positions on issues see karol2009party (2009). For a general discussion on issue advantage, see vavreck2009message (2009)). In this article, we consider the consequences of an adjudication advantage that is granted instantaneously to one of the interested parties. Following this research on political dialogue, we understand that users from different parties will engage with each other in social media prior to elections when salience is high, and the results are yet to be released. Upon adjudication, however, winners claim “ownership”, using the terminology of political dialogue theories.

While our study of electoral adjudication is informed by current research on political dialogue, two critical differences carry substantive implications. First, in presidential regimes, social media engagement takes place after the election is adjudicated. Therefore, it is unlikely to serve instrumental electoral purposes as proposed in the standard literature on political dialogue. It is unlikely that social media engagement after adjudication will be strategically directed to increase the salience of the election result. Therefore, we expect differences in Twitter engagement to result from expressive rather than instrumental behavior, as voters would not benefit electorally from increased salience after the election.

However, it is certainly possible that there are positive instrumental benefits from increased issue salience after the election when parties are called to form government shortly after (as in the United Kingdom) or when the selection of cabinet members is not solely restricted to members of the winning party or coalition. In parliamentary regimes, there are instrumental benefits from reporting on the outcome of the election. This is particularly relevant for the United Kingdom and, to a lesser degree, to the allocation of ministerial posts to allies of Jair Bolsonaro in the case of Brazil.

Our findings also offer insights into current theories of political behavior that describe how “enthusiasm” increases engagement while “anger” reduces engagement [14, 16, 17, 46]. Because there are asymmetries in “enthusiasm” and “anger” among leaders and followers, our analyses have implications for the study of network activation and content sharing after electoral adjudication. Our findings also engage with current studies of *critical events* [12, 44], which focus public attention on the consequences of an event and redefine how voters or users perceive a situation. As in the critical event theory, adjudication induces a change in dialogue that redefines the interpretation of the event (e.g., “you won because […] while I lost because”). Different from the notion of a “critical event,” salience precedes adjudication, and dialogue changes to a different extent among users that align with the winner or the loser. By contrast, critical event theory redefines the situation equally for all individuals affected by the event. By contrast, adjudication yields different interpretations of a critical event for those that win and for those that lose the election.

Finally, our theory contributes to a significant literature on the winner-loser gap [2, 5, 45], concerned with the effect of losing elections on trust in the government and satisfaction with democracy. Recent research has pointed to the importance of information for calibrating how elections shape the perceived legitimacy of democracy among losers [18]. As noted by Lelkes (2016), increases in available political information accentuate findings from the winner-loser gap scholarship. There is also research showing that voters who support the loser of an electoral contest are considerably more likely to perceive fraud than those who support the winner [47, 48]. We expand this important scholarship on electoral studies to the field of political effects of social media. Across the four elections under scrutiny in this paper, the winner-loser gap renders distinct levels of activation and engagement, and as presented by the evidence from the section on *toxic dialogue*, these reactions are driven by an increase in toxic discourse among losers and decrease among the winner group.

Extensions of the proposed model to judicial decisions, fact-checking, and sports are among the most promising future developments of a broader study of adjudication events and dialogue. In addition, future research would benefit from further study of electoral adjudication using field and experimental data in order to disentangle behavioral mechanisms behind the winner-loser gap in social media engagement.

### 6.2 Limitations of this study

There are some important limitations to this study that deserve to be noted. These limitations relate to the selection of cases, the data collection process, the choice of the dependent variable, as well as unmeasured user heterogeneity across the cases. These limitations may affect how generalizable our findings are, so let us describe each of these issues in greater detail.

To ensure that the four cases were comparable, we selected elections with two very clearly competing candidates, which concentrate most of the votes and facilitate the identification of the incumbent and opposition candidates. As described earlier in the text, the runoff elections between Macri and Fernandez in Argentina and Bolsonaro and Haddad in Brazil will likely heighten the salience of the election and raise its importance among voters. High-stakes elections in the United States and the United Kingdom should also be associated with larger differences between the winner and losers of the election. Our analyses do not test for the effect of a larger menu of candidates. So, it is likely that less polarized elections with a larger menu of candidates will also result in smaller adjudication effects. Research by [25] indicates that low information environments will be, in all likelihood, associated with smaller adjudication effects. In addition, although we select data from four different countries, we still use a small number of cases and, in particular, under a limited time frame. Therefore, generalizations from our findings should be made carefully. Future studies interested in the effects of elections on social media engagement should expand the menu of elections analyzed here.

To measure the effect of winning or losing an election, we are selecting those users classified as belonging to the two communities of the leading candidates in each country. Consequently, we are not modeling the effect of the election result among independents or among users that supported smaller non-competitive candidates. Consequently, our findings do not rule out other motivated engagement by users in the communities that are not part of our study. As an important example, users identified as Democratic or Republican supporters in the United States had a significant presence in the social media data collected during the 2019 United Kingdom election. We do not present results in this article that report on the reaction of attentive social media users that belong to communities that are foreign to each election.

There are also important limitations in the data collection process. Although Twitter provides considerable access to its internal data when compared to other social media networks, data collection is always capturing a subset of all information that may be germane to an election. Both the streaming and the resting API reported a fraction of likely data, ranging from approximately 70% of the tweets that include a vector of characters in the streaming API to less than 40% for the resting API [49]. In this study, the streaming API was only used for the candidates’ names (e.g., Macri, Fernandez, Trump, Clinton, etc.). Second, the data collected using the resting API is sensitive to internal proprietary algorithms and to previous search patterns by users. For example, recent research produced by Twitter’s Machine Learning and Fairness Team provided evidence of algorithmic bias increasing the prevalence of political content in the platform [11]. Finally, although quite prevalent in the four countries under investigation, Twitter does not represent the multitude of social media platforms in which political discussions are a central topic. Therefore, our results are constrained to Twitter users in terms of external validity.

Finally, a number of methodological decisions were made that are relevant for assessing the validity of our results. Community identification via random walk provides a good approximation to the set of users supporting the winning and losing candidate. Visual identification of the community leaders and random inspection of a sample of accounts give us confidence in the classification rule. Results using alternative community detection algorithms such as Leiden produce excellent overlap and indicate that the identification of most accounts is not sensitive to the choice of different community detection algorithms. However, it is not possible to manually validate each of the accounts in our very large networks, which generally include approximately 250,000 high-activity accounts. Similarly, the toxicity scores retrieved using the perspective API (https://perspectiveapi.com/) are subject to their own language, and algorithmic biases [50]. While our analysis measure within-country changes in toxicity at the time of the election, we do not have information to rule out biases in toxicity classifications even if language and location are held constant.

Another important limitation of our findings relates to the persistence of adjudication effects over time. Our study measures the short-term effect of adjudication shocks on the winner and losers of the election. In contrast with much of the literature on the winner-loser gap, we have no evidence of long-term differences in Twitter engagement among these different groups of voters. We have little evidence that the effect of a positive adjudication means that the winner will be more likely to mention the election result over the next few weeks or months. It may be expected that winners will be more likely to justify future policy mandates based on their election performance and that losers will appeal to other critical events unrelated to adjudication. However, we have no evidence that there are long-term engagement effects and prefer to remain agnostic as to how Twitter engagement differs between winners and losers over time.

## Supporting information

S1 File Supporting information files (Appendix). (PDF)
