# Supplementary material for: Winning! Election returns and engagement in social media
Source: PLoS One. 2023 Mar 1;18(3):e0281475. doi: 10.1371/journal.pone.0281475 (PMC9977005; doi:10.1371/journal.pone.0281475)
Supplement: S1 File — (PDF) [file pone.0281475.s001.pdf]

Winning! Election Returns and Engagement in Social Media

Supporting Information Files (SIF)

January 28, 2023

Contents

S1: Descriptive information about the "Winning!" datasets

As reported in the main paper, we conducted the data collections in all four countries using as keywords the main presidential candidates and parties (*"Clinton", "Trump", "Macri", "Fernandez", "Haddad", "Bolsonaro", "Corbyn", "Johnson", "Democratic", "Republican", "Cambiemos", "Frente de Todos", "PT", "PSL", etc.*). We supplement the data collection with the terms (*"election", "eleição", "elección"*). This procedure yield a large sample of tweets from early morning on Election Day until one day after the election (6,7 million tweets from the UK, 6,7 million tweets from Argentina, 4.9 million tweets from Brazil, and 5,2 million tweets from the US). We filtered singletons (one-time users), retain only those tweets posted in the country's primary language, and retained the first connected cluster of each country.

We filtered singletons (one-time users), retain only those tweets posted in the country's primary language, and retained the first connected cluster of each country. In all four cases, these primary connected clusters contained the main political networks that were politically engaged during the election. For community detection, we implement a two-step approach: first, we implement a computational community detection of the tweet networks via *random.walk*, assigning all accounts to unique communities (Csardi et al., 2006). Large networks with approximately 250,000 nodes impose significant constraints on the range of community detection algorithms that can be used efficiently. For the detection of non-overlapping communities, the most frequently used alternatives are the walktrap community algorithm, the Leiden algorithm, infomap, and the Luvaine algorithm. Because of the properties of a small world network such as Twitter, all four algorithms produce fairly similar and accurate community classifications (Orman et al., 2012). Differences between these algorithms are more important in networks with larger average path distances. We used the package iGraph with a default setting of 5 steps. Draws are used to connect nodes to other nodes or communities using short random

"walks." Nodes and then communities are iteratively merged together by minimizing the overall distance between nodes and communities.

Second, we sort the accounts in each community by their in-degree and the top tweets by the number of retweets and proceed to manually inspect each community to label the main political groups.

To exemplify our process, Figure 1 presents the 230,660 high-activity accounts in the primary connected network of the UK on election day. These are accounts that were either retweeted by more than two users or that tweeted about the UK election more than three times. These 230,660 accounts were responsible for 2916671/6716343 = 43% of the UK election collected on December 12, 2019.

Figure 1 shows a significant number of accounts from Republican and Democratic users in the United States, as well as activity from third parties such as the Lib-Dem and the SNP. The two communities with high-activity users that share content supporting Corbyn and Johnson included 73,657 users that deliver 707,420 tweets on December 12, 2019.

Figure 2 lists the 30 accounts with the highest in-degree in the UK, with the horizontal axes describing the number of retweets on Election day. A close examination of the top authorities provides clear evidence that there is excellent community discrimination in the data. Experts in each of the four countries will quickly recognize the key social media influencers of each community.

Further, using data collected from other projects, we compare the top authorities from these communities to the top 30 authorities of the UK election; the top 30 authorities of the Argentine election of October 27, 2019; the top 30 authorities of the Brazilian election of October 28, 2018; and the top 30 authorities of the Travel Ban protests in January of 2017 in the United States. In all four cases, community detection in these alternative datasets closely matches the ones captured on Election Night.

Finally, for validation purposes, we compare the *keyness scores* of the hashtags embed-

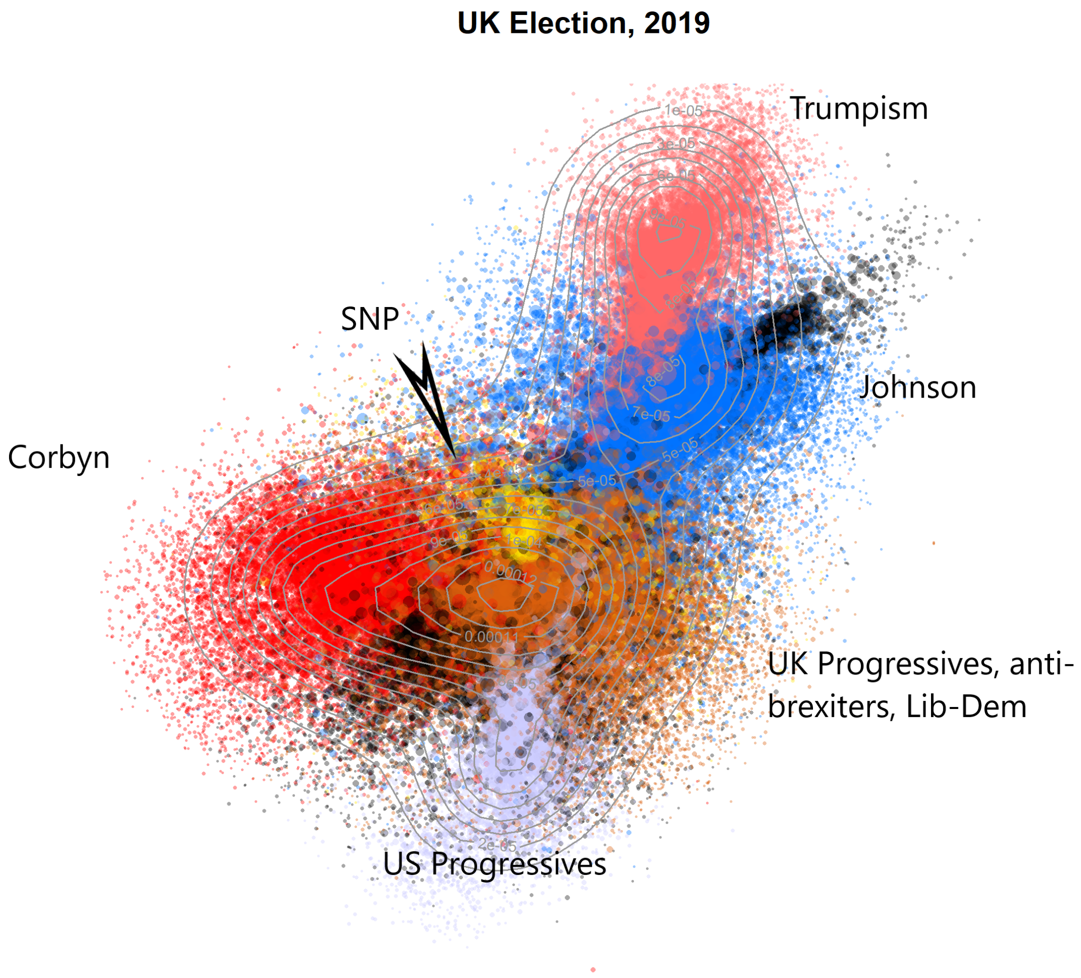

Figure 1: Election Day Network in the UK

ded in the candidates' communities and compare those features that were maximally different between these communities. "Keyness" Bondi and Scott (2010); Gabrielatos (2018) describes character strings that occur with unusual frequency. In our case, we consider as our corpus the hashtags present in the two communities of the competing candidates and rank order those hashtags that maximally discriminate between the communities. The package "Quanteda"(Benoit et al., 2018) computes the p-values of each of the features in

our corpus, in this case, the hashtags.

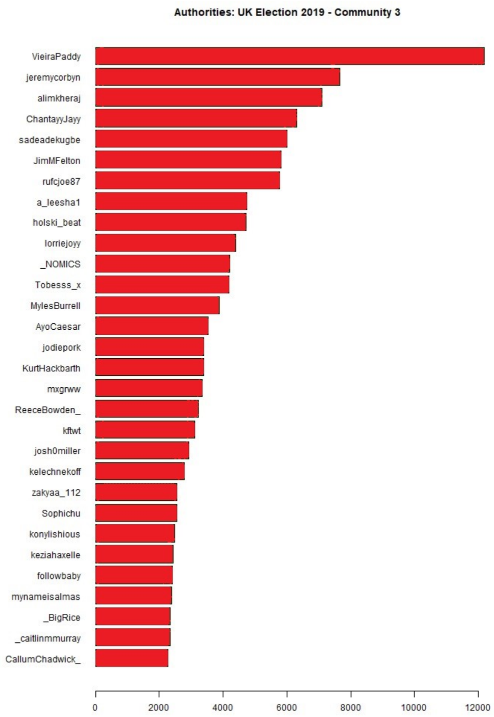

(a) Corbyn

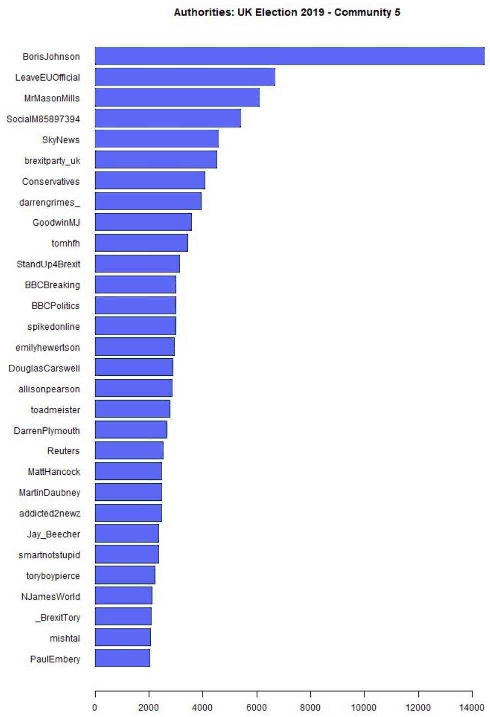

(b) Johnson

Figure 2: Leading authorities of the winning and losing communities in the United Kingdom, 2019 Election

In all four cases, we repeated the process, visually inspecting the leading authorities of each community as well as the overall distribution of the top 30 hashtags by their keyness. Figures 4 through 7 present the leading accounts of the communities in Argentina, Brazil, and the United States, each of them followed by the keyness plot of the hashtags that maximally discriminate between communities.

UK Accounts.png

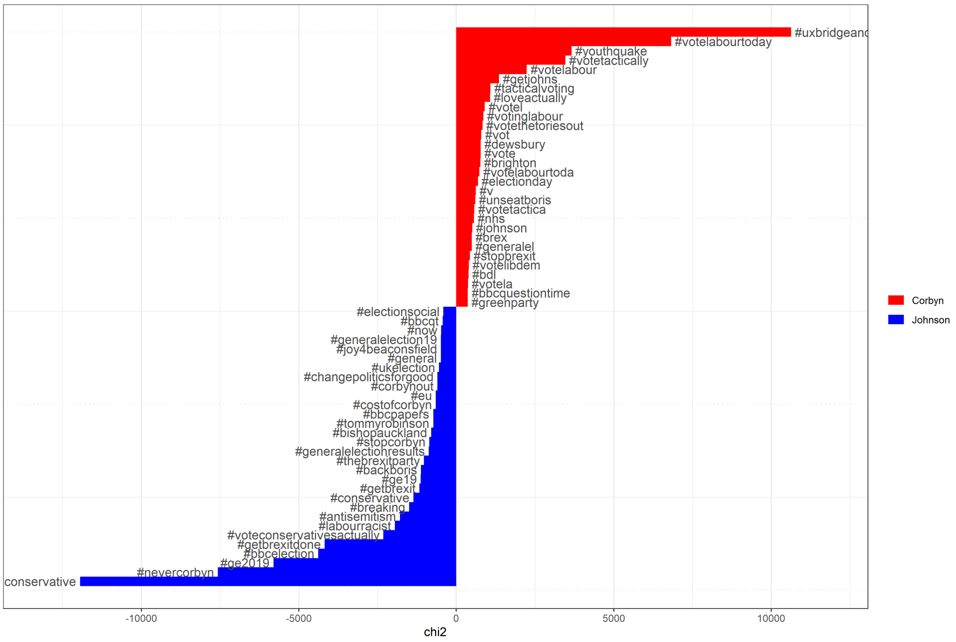

Figure 3: Selected hashtags by keyness: We visually inspect the hashtags that maximally discriminate the communities supporting Corbyn and Johnson.

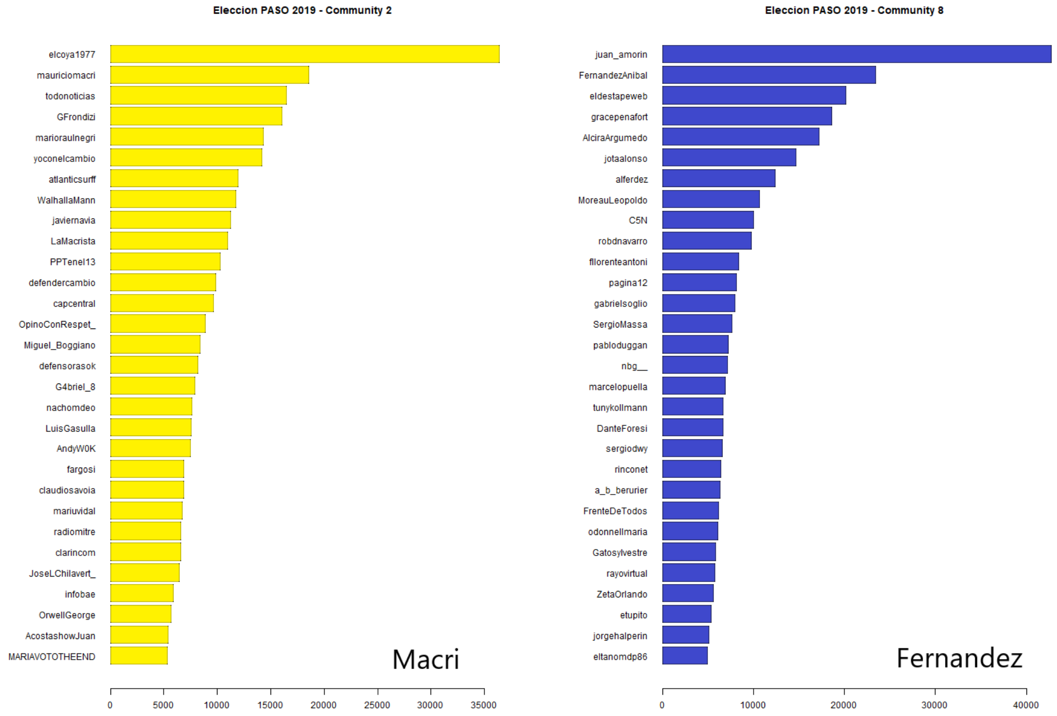

(a) Macri (b) Fernandez

Figure 4: Leading authorities of the winning and losing communities in the 2019 Election, Argentina.

Argentina Accounts.png

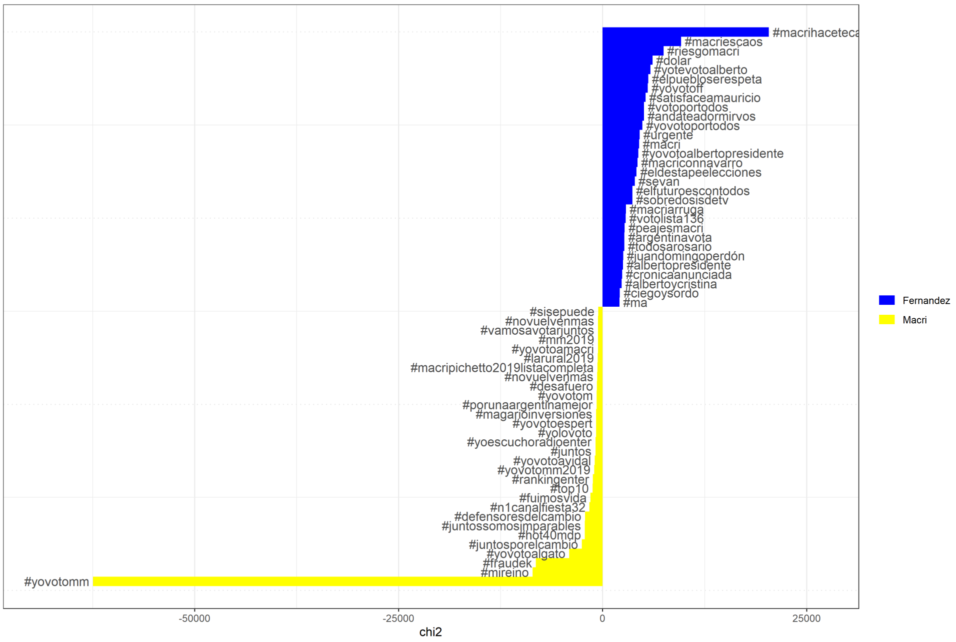

Figure 5: Selected hashtags by keyness: We visually inspect the hashtags that maximally discriminate the communities supporting Mauricio Macri and Alberto Fernandez in Argentina.

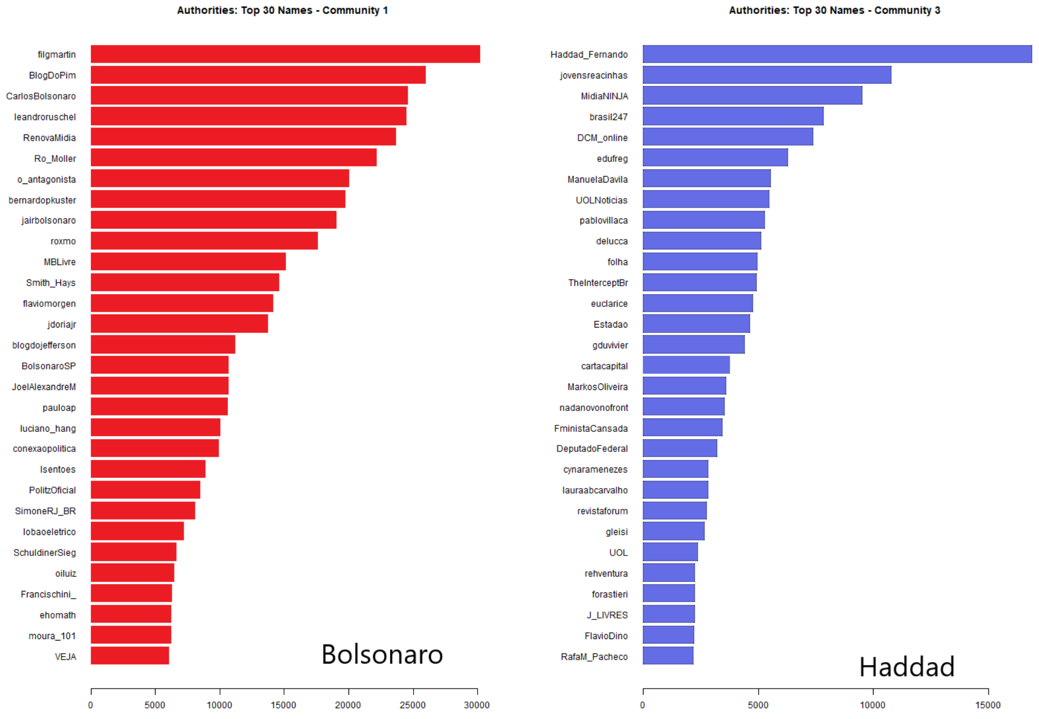

(a) Bolsonaro (b) Haddad

Figure 6: Leading authorities of the winning and losing communities in the 2018 Election, Brazil

Bolsonaro Accounts.png

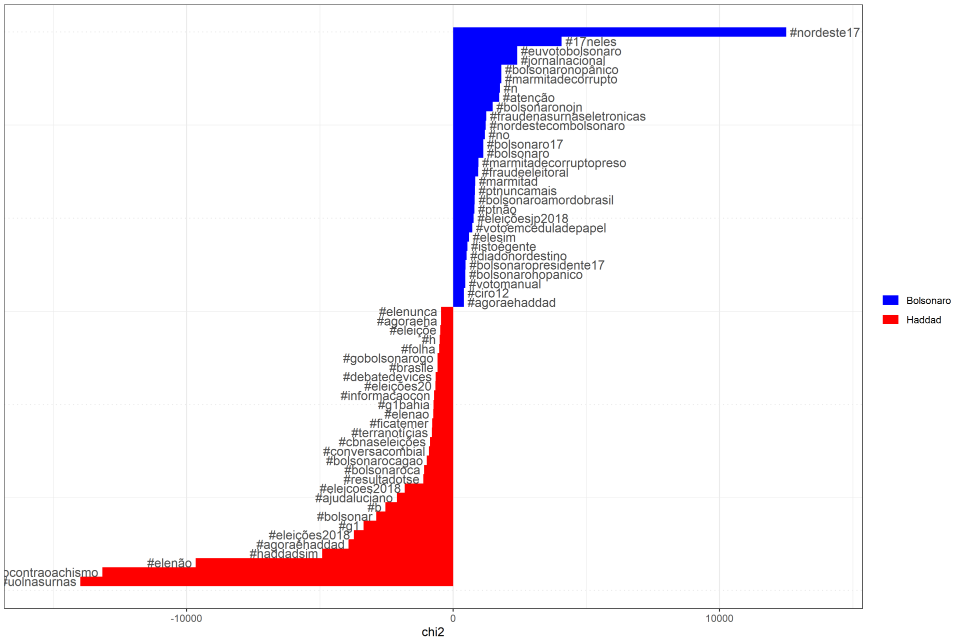

Figure 7: Selected hashtags by keyness: We visually inspect the hashtags that maximally discriminate the communities supporting Fernando Haddad and Jair Bolsonaro in Brazil.

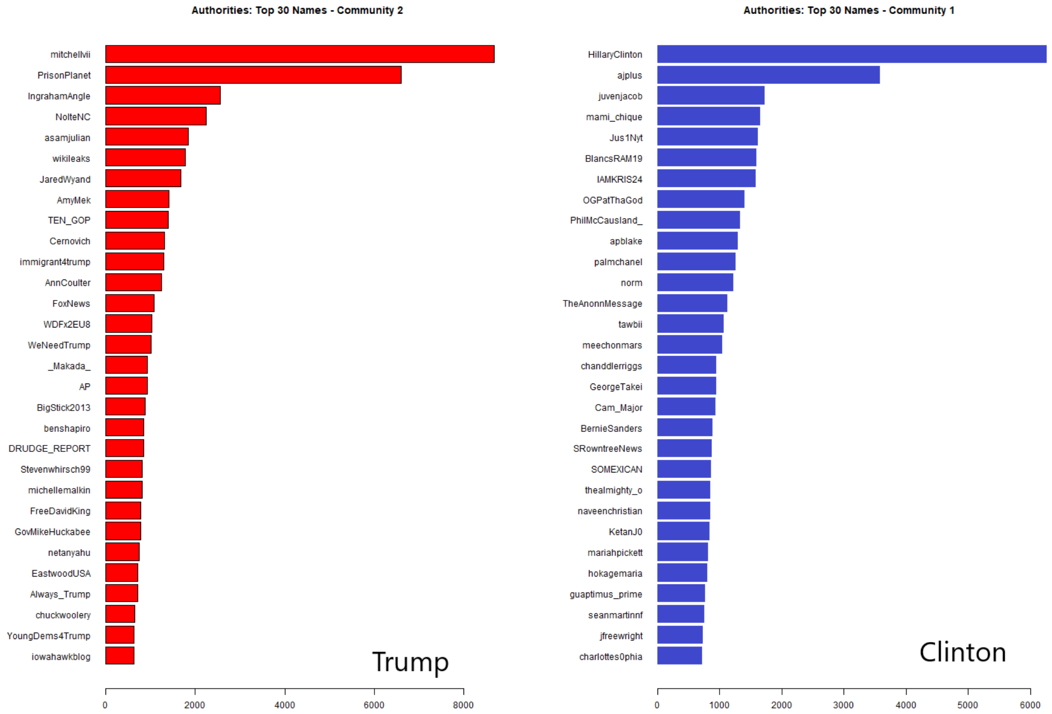

(a) Trump

(b) Clinton

Figure 8: Leading authorities of the winning and losing community in the 2016 Election, United States

US Accounts.png

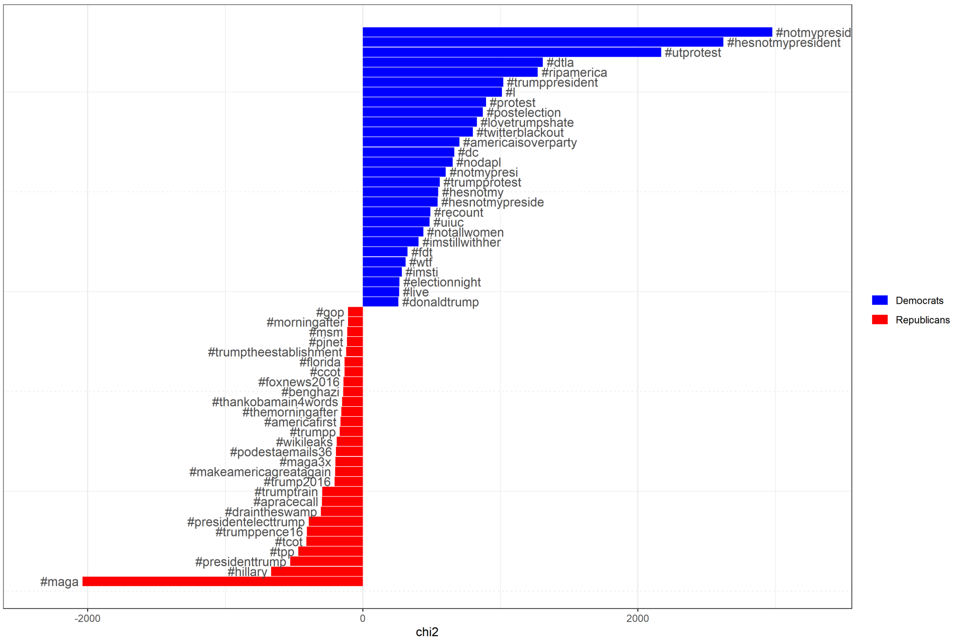

Figure 9: Selected hashtags by keyness: We visually inspect the hashtags that maximally discriminate the communities supporting Hillary Clinton and Donald Trump Jr. in the United States.

S2: Treatment effect and results using alternative bandwidths

To ensure the robustness of our findings, we present here the results using *ad-hoc* bandwidths for our main cases of the paper. We present results using intervals of five minutes from the adjudication up to two hours before and after the event. Figure 10 indicates that the results in the main paper are strongly driven by the choice of bandwidths. For all the models we report in figure 10, the vast majority of point estimates predict a decrease in time-to-retweet after adjudication – as we find in the main results of the paper. As it was reported in the paper, anticipation reduces the value of adjudication and increases information drift before the cut point.

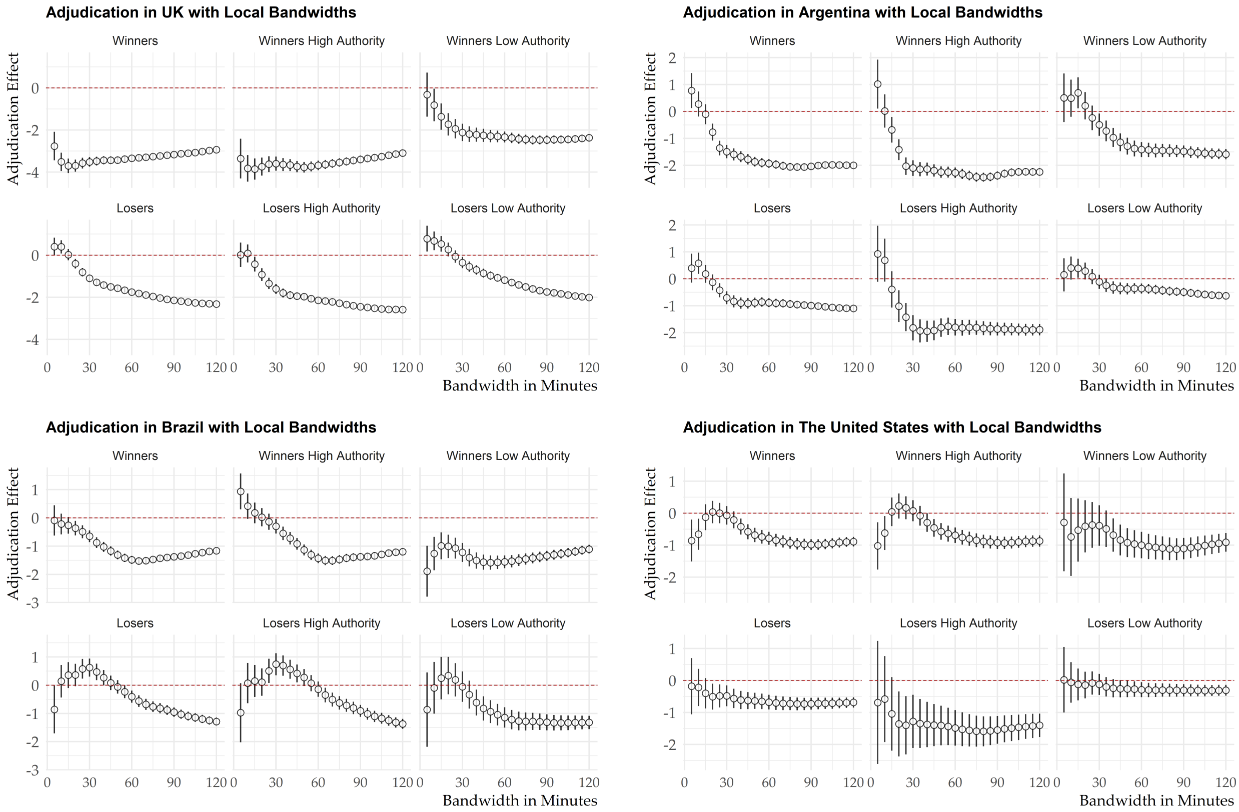

Figure 10: Adjudication in elections using local bandwidths. The y-axis plot the robust estimates and 95% confidence intervals. In the x-axis, the figures present the bandwidths

S3: Placebo Checks

In all four cases, we have direct evidence and can observe the time at which the election is adjudicated. Therefore, concerns over treatment assignment are mitigated. Additionally, since our running variable is time, sorting across the cutoff is not a concern – as it happens to be in other empirical applications using regression discontinuity designs. However, a methodological challenge with time data relates to the validity of the event adjudication compared to other similar shocks in our outcome variable over time. In this appendix, we provide placebo tests to address this potential concern.

For our test, we estimate the Adjudication model on the full set of observations - without separating the data by communities - for every ten-minute interval over six hours

in the pre-treatment data. Each interval is considered placebo cutoffs. To reduce computational costs, we use the same bandwidth of the true model to estimate the models in the placebo cutoffs – 43 minutes for the Argentinian case, 62 minutes for Brazil, 48 minutes for the United States, and 40 minutes for the UK case.

Figure 11 presents the results. The true treatment effect (using the precise time of the Event Adjudication as the cutoff point) falls well outside the null distribution of the adjudication for the four cases. In the case of the election of Donald Trump, the true effect actually overlaps with one of the tails of the placebo checks; however, the true treatment does fall outside of the 99% lower bound of the null distribution. Taken together, the placebo checks demonstrate how the adjudication treatment effects are unlikely to be driven by a random unobserved shock in the running variable.

Notice that using all the data simultaneously adds noise to the data and yields a much more conservative estimate of the true effect. This is true for each community as well as for the aggregate effect. Estimates for each community can be requested from the authors but are omitted from this SIF file for presentation purposes.

Consistent with the theory presented in the article, information drift in the United States and Brazil reduced the value of adjudication. However, placebo tests still show large and statistically significant effects that are not observed in other regions of the data.

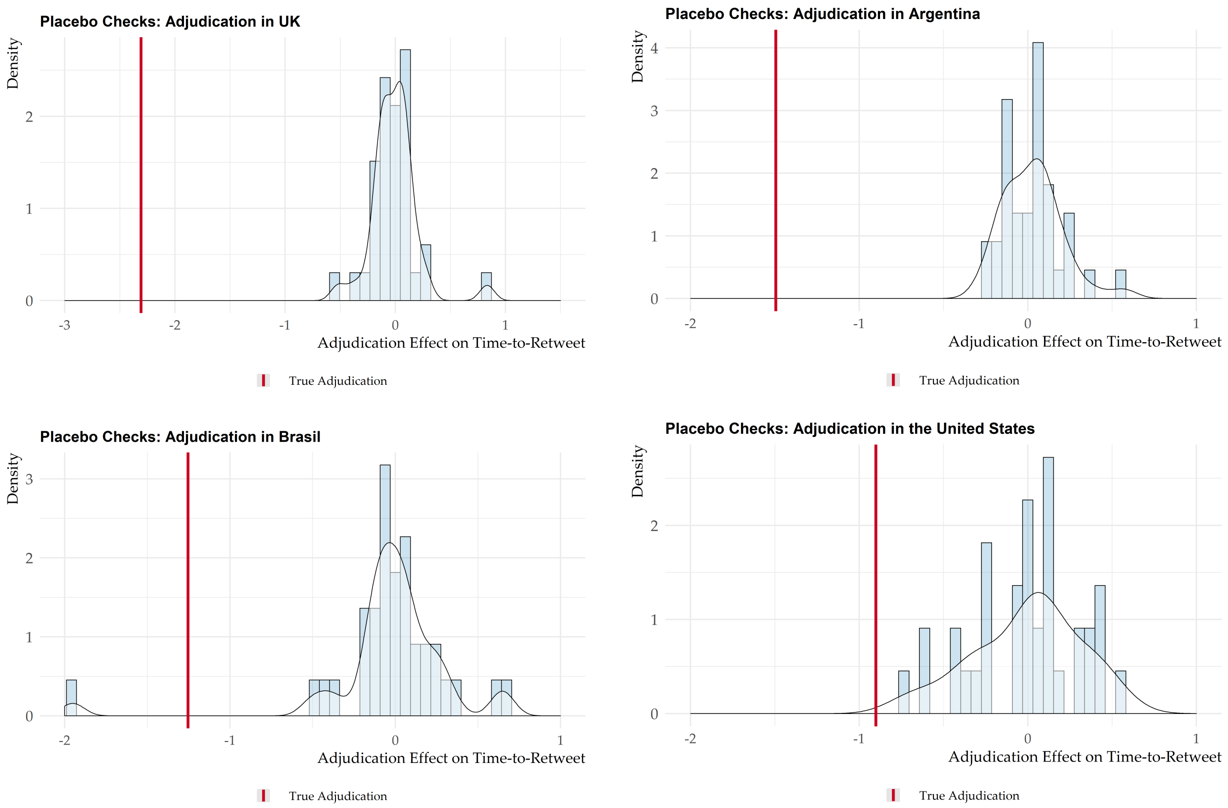

Figure 11: Placebo checks for adjudication in elections. The red lines represent the Adjudication Effect using the true cutoff. The x-axis plot the robust point estimates using placebo cutoffs every ten minutes up to 6 hours in the pre-treatment data. In the y-axis, we plot the density distribution for the adjudication effects in the null distribution.

S4: Anticipatory Behavior

As we discuss in the paper, anticipation in the adjudication is likely to occur under some conditions. For example, in the United States, the staggered release of election results allows voters to update their beliefs about the likely winners and losers of the election. For the estimation of the adjudication effects, anticipation is a threat only if the effects go counter to the expected direction of the coefficients. There are few political events that would result in social media users behaving opposite to the expected effect due to anticipation, such as fraudulent results or a very sharp change in the direction of the tally. Still, we provide a formal test of information drift that is inconsistent with the adjudication hypothesis.

We estimate the effects of adjudication using a very narrow bandwidth - every minute before the true event - for the interval of thirty minutes. By using this narrow interval in the running variable, the RD design loses the consistency of the estimator, but it gains in identifying local changes in the outcome variable. If these narrow point estimates are - on absolute terms - larger than the treatment effect, we can assume users are reducing their activity on social media before the Event Adjudication – the intercept on the left side is greater. In other words, when adjudication is larger on extremely narrow intervals, users are anticipating the effect in the opposite direction of our hypothesis, which represents a threat to the identification of our results. This pattern would be an indication that the point estimates of the main paper overestimate the effects of Event Adjudication.

Figure 12 presents the results. We do not observe anticipation leading to higher time-to-retweet in any of the four cases. If anything, in the cases of Brazil and the United States, users anticipate the results, as we expected, decreasing time-to-retweet, which indicates the effects of adjudication are likely larger than those identified in the main paper.

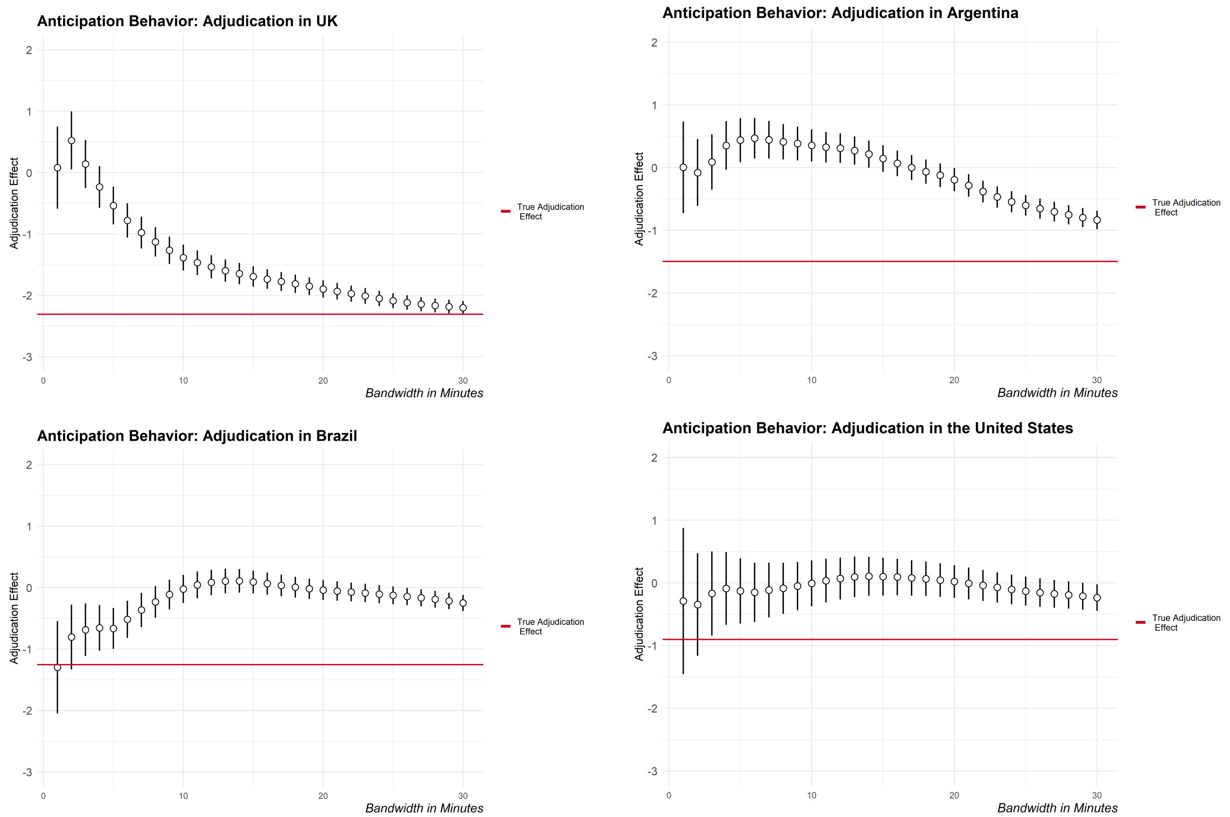

Figure 12: Anticipation of the adjudication effects. The red lines show the adjudication effect using the optimal bandwidth. The y-axis plot the robust point estimates using narrow bandwidths every minute before the Event Adjudication. If users are increasing their time-to-retweet before the event, we expect the point estimates to be larger in absolute terms compared to the true effect.

References

Benoit, K., Watanabe, K., Wang, H., Nulty, P., Obeng, A., Müller, S., and Matsuo, A. (2018). quanteda: An r package for the quantitative analysis of textual data. *Journal of Open Source Software*, 3(30):774.

Bondi, M. and Scott, M. (2010). *Keyness in texts*, volume 41. John Benjamins Publishing.

Csardi, G., Nepusz, T., et al. (2006). The igraph software package for complex network research. *InterJournal, Complex Systems*, 1695(5):1–9.

Gabrielatos, C. (2018). Keyness analysis: Nature, metrics and techniques. In *Corpus approaches to discourse*, pages 225–258. Routledge.

Orman, G. K., Labatut, V., and Cherifi, H. (2012). Comparative evaluation of community detection algorithms: a topological approach. *Journal of Statistical Mechanics: Theory and Experiment*, 2012(08):P08001.
